# Edge‐Site‐Free and Topological‐Defect‐Rich Carbon Cathode for High‐Performance Lithium‐Oxygen Batteries

**DOI:** 10.1002/advs.202300268

**Published:** 2023-04-07

**Authors:** Wei Yu, Takeharu Yoshii, Alex Aziz, Rui Tang, Zheng‐Ze Pan, Kazutoshi Inoue, Motoko Kotani, Hideki Tanaka, Eva Scholtzová, Daniel Tunega, Yuta Nishina, Kiho Nishioka, Shuji Nakanishi, Yi Zhou, Osamu Terasaki, Hirotomo Nishihara

**Affiliations:** ^1^ Advanced Institute for Materials Research (WPI‐AIMR) Tohoku University Sendai 9808577 Japan; ^2^ Institute of Multidisciplinary Research for Advanced Materials Tohoku University Sendai 9808577 Japan; ^3^ JSPS International Research Fellow (Advanced Institute for Materials Research (WPI‐AIMR) Tohoku University Sendai 9808577 Japan; ^4^ Research Initiative for Supra‐Materials (RISM) Shinshu University Nagano 3808553 Japan; ^5^ Institute of Inorganic Chemistry of Slovak Academy of Sciences Dúbravská cesta 9 Bratislava 84536 Slovakia; ^6^ Institute of Soil Research University of Natural Resources and Life Sciences Peter‐Jordan‐Strasse 82 Wien 1190 Austria; ^7^ Research Core for Interdisciplinary Sciences Okayama University 3‐1‐1 Tsushima‐Naka Kita‐ku Okayama 7008530 Japan; ^8^ Research Center for Solar Energy Chemistry Graduate School of Engineering Science Osaka University Toyonaka Osaka 5608531 Japan; ^9^ Innovative Catalysis Science Division Institute for Open and Transdisciplinary Research Initiatives (ICS‐OTRI) Osaka University Suita Osaka 5650871 Japan; ^10^ Centre for High‐Resolution Electron Microscopy (CℏEM) School of Physical Science and Technology ShanghaiTech University Shanghai 201210 China; ^11^ Shanghai Key Laboratory of High‐Resolution Electron Microscopy ShanghaiTech University Shanghai 201210 China

**Keywords:** carbon cathodes, edge sites, graphene mesosponges, lithium‐oxygen batteries, topological defects

## Abstract

The rational design of a stable and catalytic carbon cathode is crucial for the development of rechargeable lithium‐oxygen (Li—O_2_) batteries. An edge‐site‐free and topological‐defect‐rich graphene‐based material is proposed as a pure carbon cathode that drastically improves Li—O_2_ battery performance, even in the absence of extra catalysts and mediators. The proposed graphene‐based material is synthesized using the advanced template technique coupled with high‐temperature annealing at 1800 °C. The material possesses an edge‐site‐free framework and mesoporosity, which is crucial to achieve excellent electrochemical stability and an ultra‐large capacity (>6700 mAh g^−1^). Moreover, both experimental and theoretical structural characterization demonstrates the presence of a significant number of topological defects, which are non‐hexagonal carbon rings in the graphene framework. In situ isotopic electrochemical mass spectrometry and theoretical calculations reveal the unique catalysis of topological defects in the formation of amorphous Li_2_O_2_, which may be decomposed at low potential (∼ 3.6 V versus Li/Li^+^) and leads to improved cycle performance. Furthermore, a flexible electrode sheet that excludes organic binders exhibits an extremely long lifetime of up to 307 cycles (>1535 h), in the absence of solid or soluble catalysts. These findings may be used to design robust carbon cathodes for Li—O_2_ batteries.

## Graphical abstract

An edge‐site‐free and topological‐defect‐rich graphene mesosponge (GMS) is proposed as a carbon cathode for lithium‐oxygen—batteries. The GMS is highly stable, with high discharge capacity, low charge plateau and enhanced electrochemical stability compared to other commercial carbon materials. The table of contents image shows the formation of easily‐decomposable Li_2_O_2_ at the topological defects on GMS.

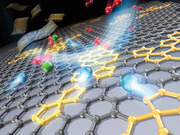

## 1 Introduction

Lithium‐oxygen (Li—O_2_) batteries with a significantly higher theoretical energy density (>3500 Wh kg^−1^) than those of conventional Li‐ion batteries are promising candidates for use in next‐generation energy storage devices.^[^ ^1^ ^]^ However, the development of stable Li—O_2_ batteries remains elusive because the batteries suffer from serious component degradation; in particular, of their carbon cathodes.^[^ ^2^ ^]^ Carbon degradation is usually caused by exposure to a severely oxidizing environment that occurs due to the presence of highly reactive oxygen species.^[^ ^3^ ^]^ Moreover, these degradation reactions become more intense at higher potentials.^[^ ^4^ ^]^ While the introduction of solid catalysts and redox mediators masks the degradation of carbon electrodes by reducing the overpotential, other problems arise, such as the decomposition of the electrolyte catalyzed by solid catalysts and Li‐anode degradation due to the shuttle effect of redox mediators.^[^ ^5^ ^]^ It is therefore crucial to improve the intrinsic stability and catalytic activity of pure carbon materials in Li—O_2_ batteries without using solid catalysts or redox mediators.^[^ ^6^ ^]^

Although various types of carbon material have been proposed as cathode materials for Li—O_2_ batteries, such as carbon nanotubes (CNT), carbon black (CB), Ketjen black (KB), activated carbons (AC), and reduced graphene oxide (rGO), their stability and catalytic activities are unsatisfactory.^[^ ^7^ ^]^ The number of carbon edge sites must be reduced to increase stability.^[^ ^8^ ^]^ However, carbon materials with a small number of edge sites, such as graphite, suffer from poor catalytic performance and large overpotentials (>4.0 V versus Li/Li^+^), leading to a variety of undesirable side reactions.^[^ ^9^ ^]^ Highly crystalline carbon materials usually have low porosities, which results in a limited capacity. The introduction of another type of catalytic site is necessary to address this trade‐off. A promising candidate is topological defects which are non‐hexagonal carbon rings formed on the graphene basal plane. Theoretical calculations have predicted that rehybridized electron orbitals of topological defects can act as active sites in Li—O_2_ batteries.^[^ ^10^ ^]^ If these active sites are embedded into a highly porous edge‐site‐free carbon framework, the resulting material should ideally demonstrate high stability, increased catalytic activity, and a large capacity. However, the fabrication of such edge‐site‐free and topological‐defect‐rich carbon materials has not yet been realized because of the interplay between these two structural features in highly porous materials. Research into the role of topological defects is therefore always affected by the presence of edge sites.

In this study, a unique 3D nanoporous graphene material, graphene mesosponge (GMS), was used as a cathode in Li—O_2_ batteries. GMS possesses developed mesoporosity and an edge‐site‐free structure, as confirmed by N_2_ adsorption/desorption and advanced temperature programmed desorption (TPD), respectively. Moreover, the topological‐defect‐rich structure of GMS is revealed here for the first time through theoretical calculations, Raman spectroscopy, and direct atomic‐resolution microscopy. Li—O_2_ batteries based on edge‐site‐free and topological‐defect‐rich GMS cathodes provide a large capacity (>6700 mAh g^−1^), low charge plateau (3.6 V [versus Li/Li^+^]) and ultra‐long cycle performance (307 cycles, >1535 h) without the aid of solid or soluble catalysts. In situ differential electrochemical mass spectrometry (DEMS), quantitative chemical titration, and density functional theory (DFT) simulations were performed to elucidate the effect of the topological defects in GMS‐based Li—O_2_ batteries. This study describes the design principles of carbon cathodes for Li—O_2_ batteries with low overpotential, excellent cycle stability, and high capacity.

## 2 Results and Discussion

### 2.1 Edge‐Site‐Free and Topological‐Defect‐Rich Carbon

Spherical Al_2_O_3_ nanoparticles (*ϕ* 9 nm, Figure S1, Supporting Information) were used as templates for the deposition of a thin carbon layer (equivalent to the thickness of a single graphene sheet) on their surface. This was performed using chemical vapor deposition (CVD) of methane at 900 °C, and through the unique catalysis of in situ‐generated O vacancies.^[^ ^11^ ^]^ The Al_2_O_3_ template was removed by hydrofluoric acid (HF) treatment, which yielded highly mesoporous carbon, denoted as carbon mesosponge (CMS). Subsequent high‐temperature annealing at 1800 °C converted the CMS into GMS. No Al signal was detected by X‐ray photoelectron spectroscopy (Figure S2, Supporting Information), and the weight loss (Figure S3, Supporting Information) of both CMS and GMS powders in the thermogravimetry analysis in the air was nearly 100%, confirming the successful removal of the Al_2_O_3_ templates. The N_2_ adsorption/desorption isotherms of CMS and GMS, as well as the reference carbons used in this study, including CNT, CB, KB, rGO, and AC, are shown in Figure S4a, Supporting Information. The type‐IV isotherms of CMS and GMS indicate their developed mesoporosities. The significant overlapping of their isotherms confirms their almost identical nanoporosities, in particular, pore‐size distributions (Figure S4b, Supporting Information), Brunauer–Emmett–Teller (BET) specific surface areas (Figure S4c, Supporting Information), and total pore volumes (Figure S4d, Supporting Information). The BET surface area of GMS (1861 m^2^ g^−1^) is comparable to that of conventional physically activated carbon (1000–2000 m^2^ g^−1^). Moreover, the GMS possesses a particularly high total pore volume (3.26 cm^3^ g^−1^), which is crucial to achieve a high capacity.

**Figure 1:**
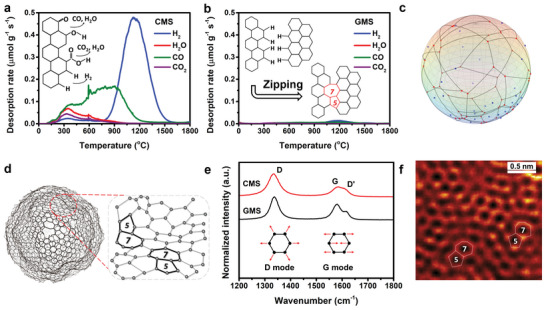
Textural properties of carbon mesosponge (CMS) and graphene mesosponge (GMS). Gas evolution patterns of H_2_, CO, CO_2_, and H_2_O during the TPD measurements for a) CMS and b) GMS. The inset of Figure 1a shows typical thermal decomposition reactions of edge sites during TPD. The inset of Figure 1b illustrates a graphene‐zipping reaction to form carbon 5‐ and 7‐membered rings. c) A mathematical model of spherical carbon with *N* = 30 base points (blue dots), Voronoi vertices (red dots), and Voronoi edges (gray curves). d) Model of GMS constructed using quenched molecular dynamics simulation. e) Raman spectra of CMS and GMS. The vibrations of D mode (A1g symmetry) and G mode (E2g symmetry) are illustrated in an inset. f) Atomic‐resolution TEM image of GMS taken at 80 kV.

Advanced TPD was used to precisely quantify (at the ppm‐level) the amount of H and O edge sites that are desorbed as H_2_, CO, CO_2_, and H_2_O gases as the sample was heated up to 1800 °C (inset of **Figure** **1**a).^[^ ^12^ ^]^ The gas evolution profile of CMS is shown in Figure 1a, and a total of 2.02 mmol g^−1^ reflects the number of carbon edge sites terminated by H and O in the CMS (Figure S5a, Supporting Information). The intense H_2_ evolution observed at 900–1600 °C corresponds to graphene‐zipping reaction^[^ ^13^ ^]^ and cannot be quantified by traditional TPD, which has an upper‐limit temperature of 1000 °C.^[^ ^8a^ ^]^ The gas evolution profile in Figure 1a indicates that thermal treatment at 1800 °C can complete the graphene‐zipping reactions to form the coalesced graphene structures of GMS (inset of Figure 1b). The GMS that was treated at 1800 °C showed only trace amounts of gas evolution, 0.09 mmol g^−1^ (Figure 1b), which was the lowest value among the reference carbon materials (Figure S5, Supporting Information). These advanced TPD analyses demonstrate the edge‐site‐free properties of the GMS. The non‐hexagonal carbon rings denoted “topological defects” could be formed when curved graphene was grown on the surface of spherical template nanoparticles during chemical vapor deposition (CVD). In addition, topological defects can form when graphene domains with different orientations coalesce during the removal of edge sites.^[^ ^14^ ^]^ Thus, GMS may be expected to contain a noticeable amount of topological defects.

In the absence of a particular method to precisely quantify the number of topological defects in a material, confirmation and quantification of their presence in the GMS framework were attempted using a combined experimental and theoretical approach. First, the number of graphene boundaries was calculated using a mathematical method, Voronoi tessellation, in which a simplified spherical model is divided into graphene *N* domains. An example with *N* = 30 is shown in Figure 1c, in which the distribution of the base points (blue), Voronoi vertices (red), and graphene domains form onto an Al_2_O_3_ nanoparticle are represented. The total length of the Voronoi edges (*L*) was then calculated for each configuration that contained a different number of base points (Figure S6, Supporting Information). Because *L* may be experimentally estimated from the TPD gas emission profile (42 nm for CMS, see Note S1, Supporting Information), the number of graphene grains may be estimated to be *N* = 3 for the GMS. A spherical unit of GMS must therefore contain a certain number of topological defects to establish a curved graphene structure. Note that an ideal sphere was used in the above representation. In contrast, the structure of the GMS should be more complex; with a number of possible graphene wrinkles forming around the Al_2_O_3_ nanoparticles. Next, the formation of “interior topological defects” was simulated using quench molecular dynamics simulations with the reaction‐state summation potential.^[^ ^15^ ^]^ The resulting structure (Figure 1d) contains many topological defects all around the curved basal plane, which eliminate the distortion of the carbon hexagonal network. From these simulations (Figure 1c,d and Figure S6, Supporting Information), the GMS is expected to exhibit topological defects both at the graphene boundaries and inside the graphene grains.

TPD measurements (Figure 1b) confirmed the edge‐site‐free structure of GMS. Raman spectroscopy was used as an indicator of defect density (Figure 1e).^[^ ^16^ ^]^ To simulate the characteristic (D, G, and D′) bands present in the Raman spectrum of the GMS, DFT calculations were performed using the Vienna Ab Initio Simulation Package (VASP).^[^ ^17^ ^]^ The calculated vibration modes of graphene with topological defects and perfect graphene are shown in Figure S7, Supporting Information. The simulated vibrations at 1385, 1625, and 1642 cm^−1^ correspond to the experimentally observed D, G, and D′ peaks of the GMS, respectively.^[^ ^18^ ^]^ In general, in‐plane breathing vibrations of the aromatic ring structure (A_1g_ symmetry) become active near graphene defects at which the symmetry of perfect graphene is broken; for example, at carbon edge sites and topological defects. These results suggest that the D‐band in edge‐site‐free GMS originates in the in‐plane breathing vibrations near topological defects (Figure S7, Supporting Information). The intensity ratio of the D and G band (*I*_D_/*I*_G_ ratio) (1.45) is greater than that of the reference carbon (0.87–1.30, Figure S8a, Supporting Information). The relationship between the *I*_D_/*I*_G_ ratio and the mean distance between defects (*L*_D_) was plotted using the relationship reported in previous literature (Figure S8b and Note S2, Supporting Information).^[^ ^16^, ^19^ ^]^ The smaller the distance between defects (*L*_D_), the greater the defect density and probability that the defects include both edge sites and topological defects. However, in the case of edge‐site‐free GMS, the *L*_D_ value results only from the presence of topological defects (inset of Figure S8b, Supporting Information).

Although the removal of edge‐sites leads to a reduction in the *I*_D_/*I*_G_ ratio from 2.33 (CMS) to 1.45 (GMS) and a corresponding increase in *L*_D_, the *L*_D_ of the GMS is still lower than those of rGO, KB, CB, and CNT, indicating its topological‐defect‐rich structure. Furthermore, we employed atomic‐resolution transmission electron microscopy (TEM) to directly observe topological defects in the GMS. Many 5‐ and 7‐membered rings were identified throughout the structure (Figure 1f) and at grain boundaries (Figure S9, Supporting Information). Both theoretical and experimental methods therefore confirmed the topological‐defect‐rich nature of the GMS surface, which was expected to function as a catalytic active site.

### 2.2 Electrochemical Performance in Li‐O_2_ Batteries

**Figure 2:**
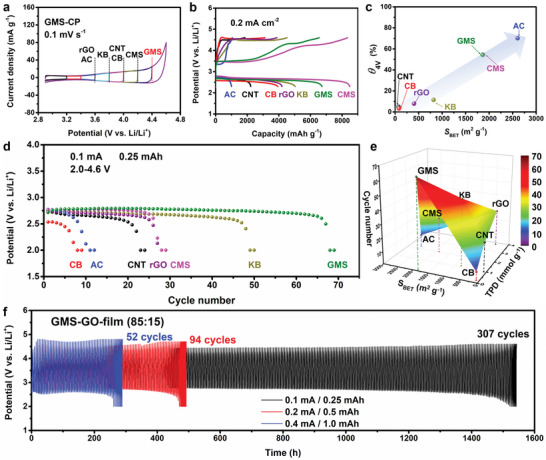
Electrochemical measurements obtained using a coin cell in an O_2_‐saturated 0.5 m LiTFSI/TEGDME. a) CV patterns of the GMS‐CP cathode only toward the positive‐potential direction. For reference, the onset potential of anodic oxidation in each carbon material is described. b) Galvanostatic full‐discharge‐charge curves of GMS‐CP and reference‐carbons‐based Li—O_2_ batteries. c) A plot of charge–capacity ratio under 4.0 V (*θ*_4 V_) against BET surface area. d) Discharge potentials and cycle performance of Li—O_2_ batteries with different carbon cathodes. e) Relationship between the physicochemical properties of carbon materials and the cycle performance of Li—O_2_ batteries. f) Long cycle performance of GMS‐GO‐film under different currents and capacities.

The electrochemical oxidation resistance of edge‐site‐free and topological‐defect‐rich GMS in O_2_ was first examined in a typical electrolyte, 0.5 m lithium bis(trifluoromethanesulfonyl)imide (LiTFSI) dissolved in tetraethylene glycol dimethyl ether (TEGDME) using a 2032 coin cell, and in the absence of Li_2_O_2_. In particular, a mixture of GMS powder and a polymer binder was attached to carbon paper (CP) to form a cathode (denoted GMS‐CP). Cyclic voltammetry (CV) was performed with a fixed lower potential of 2.9 V and stepwise expansion of the upper‐limit potential from 3.2 to 4.6 V (versus Li/Li^+^) for GMS‐CP (**Figure** **2**a) and the reference carbon cathodes (Figure S10, Supporting Information). The average carbon loading amount is 0.7 mg cm^−2^. Apart from the double‐layer capacitance that depends on their specific surface area (Figure S4c, Supporting Information), all the carbons exhibited a hump of anodic current that corresponded to degradation reactions. The onset potentials of reference carbons are indicated in Figure 2a. The CMS showed better stability (onset potential of 4.2 V) than the other conventional carbons, despite the presence of a relatively high number of edge sites (Figure S5a, Supporting Information). This suggests that the specific nanobubble‐like mesoporous framework was relatively stable. Moreover, the proposed GMS‐CP demonstrates better stability than CMS, as evidenced by an extended onset potential of up to 4.4 V. As the GMS possesses porous frameworks identical to those of CMS (Figure S4, Supporting Information), this stability enhancement may be ascribed to the edge‐site‐free nature of GMS. The excellent stability of GMS also indicates that its topological‐defect‐rich nature does not lower its electrochemical oxidation resistance in an O_2_‐containing electrolyte.

Next, CV was performed with a lower limited potential extended to 2.0 V (Figure S11, Supporting Information). The CV curves show a cathodic peak and counterpart anodic peaks, which correspond to Li_2_O_2_ formation and decomposition, respectively. Full‐discharge‐charge tests were then performed within the cut‐off potential of 2.3–4.6 V (versus Li/Li^+^), as shown in Figure 2b. The CMS‐CP and GMS‐CP demonstrate large discharge capacities of up to 8475 and 6727 mAh g^−1^, respectively. During the charging process, AC‐CP, CMS‐CP, and GMS‐CP show a charge plateau under 3.6 V. This is consistent with the appearance of anodic peaks at ≈3.6 V, as shown in the CV curves(Figure S11e–g, Supporting Information). The low potential charge plateau indicates the formation of discharge products that can be easily decomposed.^[^ ^20^ ^]^ For CNT‐CP and CB‐CP, the charge potential increases rapidly to over 4.4 V at an early stage in the charging process. The charge–capacity ratio under 4.0 V (*θ*_4 V_) in the full‐discharge‐charge test represents the proportion of the easily decomposable part of the discharge products formed during discharging. The *θ*_4 V_ value therefore can be used to evaluate the catalytic effect of different carbons (Figure S12, Supporting Information).^[^ ^8^, ^21^ ^]^ As shown in Figure 2c, *θ*_4 V_ is positively correlated with the BET specific surface area (*S*_BET_). More importantly, the *θ*_4 V_ values of CMS and GMS are overlapped, which suggests that the number of active sites at edge sites in CMS is the same as the number of active sites at topological defects formed by the zipping of edge sites in GMS.

Galvanostatic discharge–charge tests with a limited capacity of 0.25 mAh and a current of 0.1 mA were performed to evaluate the cycle stability of the carbon cathodes (Figure S13, Supporting Information), and the results are summarized in Figure 2d. The edge‐site‐free and topological‐defect‐rich GMS‐CP cathode exhibited the longest cycle life, with far superior properties to the other cathodes. The cycle numbers of the Li—O_2_ batteries are plotted against TPD total gas evolution and *S*_BET_, as shown in Figure 2e. From our experimental results, we conclude that a promising carbon cathode for Li—O_2_ batteries must simultaneously meet two requirements: a minimal number of edge sites and a sufficient number of active sites (high surface area). It is widely believed that a low number of edge sites ensure the stability of carbon.^[^ ^8b,c^ ^]^ However, the removal of edge sites or functional groups is usually accompanied by the elimination of active sites in carbon, which results in poor battery performance characteristics, such as a large overpotential.^[^ ^8a^ ^]^ It is therefore difficult to achieve both a low charge plateau and good cycle stability using conventional carbon‐based materials.^[^ ^22^ ^]^ Note that Li—O_2_ batteries based on pure carbon materials without solid catalysts are discussed here. Although loading catalysts on carbon helps to reduce the overpotential, the problem of facilitated decomposition of electrolytes by the catalysts remains. This implies that GMS is a unique and promising carbon cathode for use in Li—O_2_ batteries; providing: 1) superior stability owing to its edge‐site‐free property; 2) good catalytic performance owing to its topological‐defect‐rich nature; and 3) a large capacity due to its developed mesoporosity.

To further improve the cycle stability of the proposed GMS‐based cathode, graphene oxide (GO) was used as an inorganic binder to replace the unstable organic PVDF binder.^[^ ^23^ ^]^ A free‐standing and flexible GMS‐GO‐film was prepared using a simple filtration process (Figure S14, Supporting Information). As shown in Figure S15, Supporting Information, the GMS‐GO‐films fabricated with two different GMS:GO ratios (80:20 and 85:15) retained type‐IV N_2_ adsorption/desorption isotherms. As intended, the GMS‐GO‐films demonstrated better anodic stabilities than GMS‐CP (Figure S16a, Supporting Information), along with the unique catalysis that induces low‐potential charge (Figures S16b and S17, Supporting Information). As shown in Figure S17, Supporting Information, a lower GO content is advantageous for better porosity and larger capacity. However, it was not possible to fabricate a mechanically rigid film at GO content below 15%. The cycle performance of the Li—O_2_ battery based on the best GMS‐GO‐film (85:15) cathode is shown in Figure 2f. At 0.1 mA and 0.25 mAh, the GMS‐GO‐film exhibits super‐stable cycle performance over 307 cycles (1535 h). This is one of the best cycle performances recorded for Li—O_2_ batteries with a pure carbon cathode, without a solid catalyst nor redox mediator (Table S1, Supporting Information).^[^ ^2^, ^6^ ^]^ Moreover, the proposed GMS‐GO‐film provides good performance at higher current densities (0.2 and 0.4 mA) and larger cut‐off limited capacities (0.5 and 1.0 mAh). The good rate performance of GMS‐GO‐film with a limited capacity of 0.5 mAh also demonstrates the superior stability of an all‐carbon electrode composed of GO and GMS (Figure S18, Supporting Information). The results obtained using the proposed GMS‐GO‐film indicate the importance of binders, aside from the stability of the carbon powder. The binder was not optimized here because developing a binder was not the focus of this study.

### 2.3 Discharge‐Charge Mechanism of the GMS Cathode

**Figure 3:**
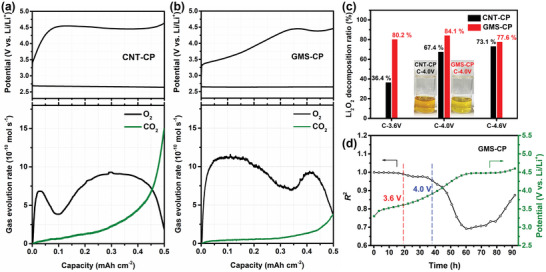
Quantification of Li_2_O_2_ during discharge–charge process. Galvanostatic discharge–charge curves and gas evolution rate during the charging process of Li—O_2_ batteries in in situ DEMS tests with a) CNT‐CP and b) GMS‐CP. c) Li_2_O_2_ decomposition ratios in cathodes charged to different potentials. These values were calculated as a ratio of the lost Li_2_O_2_ amount (determined from the chemical titration) to the estimated amount of Li_2_O_2_ deposited from the discharge capacity. Insets of (c) show the digital photos of TiOSO_4_ solutions reacted with CNT‐CP and GMS‐CP cathodes at C‐4.0 V. d) R^2^ values calculated from the charge potential change *E* for each step in GITT, plotted as a function of *t* ^1/2^. GITT curves of Li—O_2_ batteries with a current density of 0.2 mA cm^−2^ for 1.0 mAh cm^−2^ and a 180‐min time interval during the charging process.

The ex situ X‐ray diffraction (XRD) patterns of GMS‐CP and reference CNT‐CP (Figure S19, Supporting Information) confirm that the formation and decomposition of Li_2_O_2_ dominate the discharge–charge reaction.^[^ ^2^ ^]^ Thus, the basic reactions of GMS are similar to these of typical carbon cathodes. However, in situ DEMS revealed a unique reaction process of GMS‐CP. The reference CNT‐CP cathode followed a two‐step O_2_ evolution during charging (**Figure** **3**a): a small peak between 0 and 0.1 mAh cm^−2^, followed by a broad peak that corresponded to Li_2_O_2_ decomposition at a low potential, and a high plateau potential at ≈4.5 V. In comparison, the GMS‐CP cathode showed a large and broad O_2_ evolution peak between 0 and 0.35 mAh cm^−2^, followed by a small peak (Figure 3b). For both samples, the first O_2_ evolution peak may be ascribed to the oxidation of the easily decomposable discharge products.^[^ ^4^, ^20^, ^24^ ^]^ The second O_2_ evolution peak at a higher potential may be ascribed to the decomposition of crystalline Li_2_O_2_.^[^ ^4^, ^25^ ^]^ In GMS‐CP, the charging process was mainly occupied by the first reaction at a lower potential, which was beneficial for protecting the cathode from severe oxidation.^[^ ^7^, ^8^ ^]^ The CO_2_ evolution rate of GMS‐CP was always lower than that of CNT‐CP during the whole charging process, which proves the excellent stability of the GMS (Figure 3a,b).^[^ ^9^ ^]^ In addition, CMS‐CP exhibits similar charge plateaus, resulting in similar gas evolution curves as GMS‐CP during the first charge process (Figure S20, Supporting Information), possibly due to their similar mesoporosity (Figure S4, Supporting Information).

To quantify Li_2_O_2_ decomposition, chemical titration measurements were performed to monitor the color change due to the presence of TiO_2_SO_4_, which was formed from the chemical reaction of the Li_2_O_2_ and the TiOSO_4_ solution (Figure S21, Supporting Information).^[^ ^26^ ^]^ Four CNT‐CP and four GMS‐CP based Li—O_2_ batteries were assembled, and discharge–charge tests were stopped at different points: discharge to 1.0 mAh (D‐1.0mAh), then charge to 3.6 (C‐3.6 V), 4.0 (C‐4.0 V) and 4.6 V (C‐4.6 V) (Figure S22, Supporting Information). After these tests, four CNT‐CP and four GMS‐CP cathodes were removed from the batteries and subjected to chemical titration. The color change of the solution from dark yellow to transparent, which indicated the Li_2_O_2_ decomposition as the charging process progressed, is shown in Figure S23, Supporting Information. The theoretical and measured weights of the residual Li_2_O_2_ on the after‐charge cathodes are summarized in Tables S2 and S3, Supporting Information. As shown in Figure 3c, the Li_2_O_2_ decomposition ratios in GMS‐CP were always higher than those in CNT‐CP. This is evident from the different colors of the solutions, in particular, those of samples C‐3.6 V and C‐4.0 V, which correspond to higher O_2_ and lower CO_2_ evolution rates for GMS‐CP compared to CNT‐CP under 4.0 V in the DEMS tests (Figure 3a,b).

The galvanostatic intermittent titration technique (GITT) was used to investigate the kinetic charge process on GMS‐CP (Figure S24 and Note S3, Supporting Information). According to the literature,^[^ ^27^ ^]^ Li‐ion conduction in Li_2_O_2_ can be calculated based on the relationship between the potential (*E*) and the square root of time (*t* ^1/2^), as shown in Equation (1):

(1) $$D_{\mathrm{Li}}=\frac{4}{\pi }\;{\left(\frac{mV_{M}}{MS}\right)}^{2}{\left(\frac{\Delta E_{S}}{\tau \left(\frac{dE}{dt^{\frac{1}{2}}}\right)}\right)}^{2}$$

where *m*, *V*_M_, *M*, and *S* represent the mass, molar volume, molecular weight of Li_2_O_2_ and electrode surface area, respectively. Representative plots of *E* versus *t* ^1/2^ for the different periods of the GITT tests, and the corresponding squared correlation coefficient (R^2^) for each period, are shown in Figure S25, Supporting Information. Previous studies reported that the coefficient of determination (R^2^) is related to the Li_2_O_2_ decomposition mechanism.^[^ ^4^, ^28^ ^]^ A R^2^ close to 1.0, which indicated a good linear relationship between *E* and *t* ^1/2^, suggests that the Li_2_O_2_ decomposition process is dominated by a solid‐solution mechanism in which Li^+^ ions diffuse according to Fick's law of diffusion (Figure S25a, Supporting Information).^[^ ^25c^ ^]^ This solid‐solution mechanism begins with the delithiation of Li_2_O_2_ (Li_2_O_2_ → *x*Li^+^ + *x*e^−^ + Li_2−_ *_x_* O_2_), followed by the continuous delithiation of the Li‐depleted Li_2−_ *_x_* O_2_ phase. In contrast, the fact that *E* and *t* ^1/2^ are uncorrelated (R^2^ ≪ 1.0) indicates the phase separation of Li_2−x_O_2_ (Figure S25b,c, Supporting Information).^[^ ^4a^ ^]^ The variation in R^2^ and potentials as a function of time throughout the GITT tests are shown in Figure 3d. Below 3.6 V, the R^2^ values are quite close to 1.0, which indicates a decomposition process dominated by a solid‐solution mechanism. Considering the high O_2_ evolution rate in the DEMS test (Figure 3b) and the high Li_2_O_2_ decomposition ratio (Figure 3c), the charge reaction below 3.6 V should correspond to the decomposition of easily decomposable Li_2_O_2_.^[^ ^20^, ^29^ ^]^ With the consumption of these easily decomposable discharge products, the R^2^ values gradually deviate from 1.0, and even drop to 0.7. This phenomenon indicates that the decomposition mechanism of Li_2_O_2_ may change from that of a solid‐solution to a two‐phase type.^[^ ^28^ ^]^

### 2.4 Easily Decomposable Li_2_O_2_ in GMS

To further clarify the decomposition mechanism of Li_2_O_2_ on GMS, ^18^O_2_/^16^O_2_ isotope DEMS tests were conducted. Discharge was performed with ^18^O_2_/^16^O_2_ (^18^O_2_ first and then ^16^O_2_, Figure S26a, Supporting Information) and ^16^O_2_/^18^O_2_ supply (^16^O_2_ first and then ^18^O_2_, Figure S26b, Supporting Information). In both cases, the second oxygen isotope that was supplied was detected as the corresponding O_2_ at the beginning of charge process. Moreover, an extremely small amount of ^16^O^18^O was detected during the charging process, which proves that most O—O bonds were not cleaved.^[^ ^30^ ^]^

**Figure 4:**
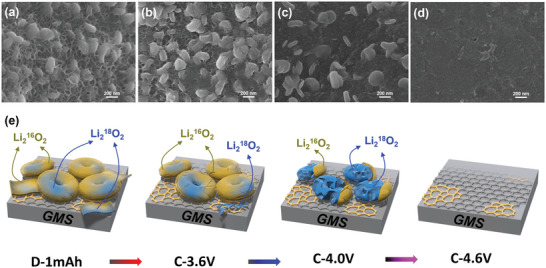
Morphology of Li_2_O_2_ during discharge–charge process. SEM images of four GMS‐CP cathodes under different discharge–charge conditions taken at the electron impact energy of 5.0 keV, after discharging to a) 1 mAh, and after charging to b) 3.6 V c) 4.0 V, and d) 1 mAh. e) Proposed charge mechanism for Li—O_2_ battery with GMS‐CP cathode.

The morphological changes of Li_2_O_2_ during the charging process were shown in scanning electron microscope (SEM) images (**Figure** **4**a–d), and the possible mechanism of these changes is summarized in Figure 4e. After discharge to a limited capacity of 1 mAh, floc‐like Li_2_O_2_ nanosheets appeared on the GMS, in addition to the typical Li_2_O_2_ toroids (Figure 4a). The floc‐like nanosheets partially disappear at 3.6 V (Figure 4b) and completely disappear at 4.0 V (Figure 4c). This implies that the Li_2_O_2_ nanosheets decomposed more easily at low charge potentials. However, the Li_2_O_2_ toroid particle size slightly decreased at 4.0 V (Figure 4c), and these disappeared at 4.6 V (Figure 4d). This indicates that the toroids were composed of crystalline Li_2_O_2_, and correspond to a charge plateau at ≈4.5 V. SEM images of GMS‐CP cathodes after galvanostatic full‐discharge‐charge tests show a similar Li_2_O_2_ formation and decomposition phenomenon (Figure S27, Supporting Information). Both Li_2_O_2_ nanosheets and Li_2_O_2_ toroids appeared on the after‐full‐discharge cathode, and most of the Li_2_O_2_ nanosheets were decomposed after charging back to 3.6 V (versus Li/Li^+^).

The proposed Li_2_O_2_ decomposition mechanism during the charging process is shown in Figure 4e. For discharge with an ^18^O_2_/^16^O_2_ supply (Figure S26a, Supporting Information), the Li_2_O_2_ nanosheets and possible Li‐depleted (Li_2−_ *_x_* O_2_) phase on the outer layer of crystalline Li_2_O_2_ toroids (which may have formed at a later stage of discharge process under an ^16^O_2_ atmosphere) should have decomposed first, via a solid‐solution mechanism. This would lead to a higher ^16^O_2_ evolution rate in Figure S26a, Supporting Information. The disappearance of the easily decomposable Li_2_O_2_ nanosheets and non‐stoichiometric Li_2−x_O_2_ led to an increased charge potential and a reduced O_2_ evolution rate (Figure S26a, Supporting Information).^[^ ^4a^ ^]^ For C‐4.0 V, the ^16^O‐rich outer layer of the Li_2_O_2_ toroids was exhausted, and the ^18^O‐rich inner crystals that were newly exposed to the electrolyte gradually began to decompose. Finally, all the ^18^O‐rich Li_2_O_2_ crystals are decomposed under a high potential charge via a two‐phase mechanism, resulting in a higher ^18^O_2_ evolution rate than ^16^O_2_ (Figure S26a, Supporting Information).

**Figure 5:**
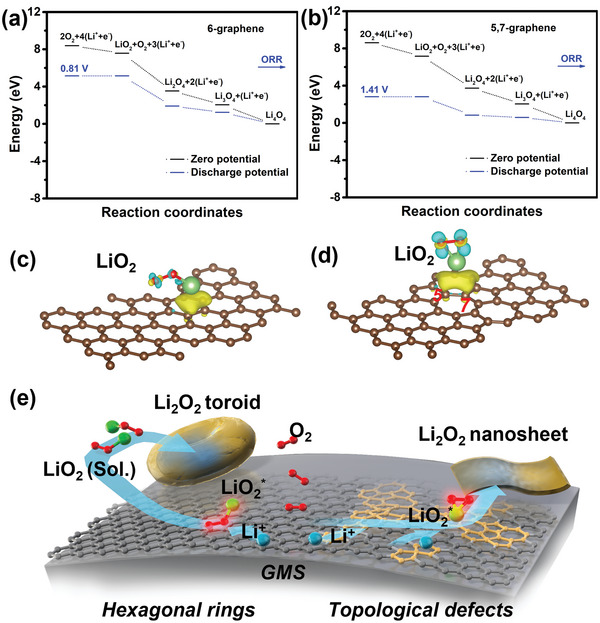
DFT calculations and proposed mechanism for Li_2_O_2_ formation. Calculated free energy diagram for the ORR/OER process on a) 6‐graphene and b) 5,7‐graphene models. Charge difference plots of LiO_2_ adsorbed on c) 6‐graphene and d) 5,7‐graphene. e) Schematic illustrations of discharge mechanism for GMS cathodes.

The formation mechanism of the easily decomposable Li_2_O_2_ was also investigated. Because GMS is an edge‐site‐free material, the remaining possibilities of catalytic sites are topological defects. Thus, DFT calculations were performed on two models (Figure S28, Supporting Information): pristine hexagonal graphene (6‐graphene) and graphene with topological defects containing 5‐ and 7‐membered rings (5,7‐graphene). Compared to 6‐graphene, the presence of topological defects in 5,7‐graphene leads to the accumulation of electron density around the topological defects, and depletion at the center of the heptagonal rings (Figure S28d, Supporting Information). The energy of Li_4_O_4_, a dimer of the Li_2_O_2_ solid, was then calculated on both the pristine and defective graphene surfaces using DFT (Figure S29, Supporting Information). **Figure** **5**a,b shows the calculated energy diagrams at different overpotentials: zero potential and discharge potential, which correspond to Li_4_O_4_ formation via oxygen‐reduction reaction (ORR). The rate‐determining step (RDS) is the formation of LiO_2_, which is O_2_ → LiO_2_ for the ORR. The charge density difference between LiO_2_ adsorbed on 6‐graphene and 5,7‐graphene is shown in Figure 5c,d. The yellow region (charge accumulation) represents the adsorption affinity of LiO_2_ on the carbon surface, where the electron density from Li is attracted to the carbon surface. The adsorption affinity of LiO_2_ for topological defects was stronger than that at the hexagonal rings. This is similar to the O_2_ affinity, which indicates a shorter adsorption distance and larger charge depletion region (blue color) on graphene with topological defects (Figure S30, Supporting Information). The DFT calculations therefore suggest that topological defects on GMS affect the adsorption affinities of O_2_ and LiO_2_ (Table S4, Supporting Information).

Based on our DFT calculations and experimental results, a possible reaction mechanism for Li_2_O_2_ formation on edge‐site‐free and topological‐defect‐rich GMS is proposed in Figure 5e. First, O_2_ is adsorbed on the active site of the GMS, where a one‐electron transfer reaction (O_2_ + Li^+^ + e^−^ → LiO_2_) occurs, and adsorbed LiO_2_ (LiO_2_^*^) is formed at the O_2_/Li^+^/e^−^ three‐phase interface.^[^ ^2b^ ^]^ Owing to the different adsorption affinity of LiO_2_^*^ on hexagonal rings and topological defects, two different Li_2_O_2_ formation mechanisms occur in GMS‐based Li—O_2_ batteries. In the region that contains only hexagonal rings, the adsorption affinity of LiO_2_^*^ is relatively low. It is more likely that LiO_2_^*^ dissolves in the liquid electrolyte solution to form LiO_2_ (Sol.). In this case, Li_2_O_2_ toroids are formed by a solution mechanism dominated by a disproportionation reaction (LiO_2 (Sol.)_ + LiO_2 (Sol.)_ → Li_2_O_2_ + O_2_).^[^ ^31^ ^]^ In contrast, a second electron transfer reaction (LiO_2_^*^ + Li^+^ + e^−^ → Li_2_O_2_) is more likely to occur on the carbon surface that contains topological defects because the affinity of LiO_2_^*^ is stronger at these active sites. This surface mechanism leads to the formation of floc‐like nanosheets, which easily decompose at a low charge plateau.^[^ ^20^, ^32^ ^]^ Topological defects at the GMS basal plane therefore tailor the morphology of the discharge products in Li—O_2_ batteries. While some studies have reported the formation of easily decomposable Li_2_O_2_ by nanopores^[^ ^20^, ^22^ ^]^ and oxygen‐functional groups,^[^ ^8a,b^ ^]^ the experimental and theoretical investigations described here indicate that the catalytic sites in GMS are topological defects.

## 3 Conclusion

In this study, GMS is proposed as a carbon cathode for Li—O_2_ batteries. Comprehensive experimental and theoretical characterizations confirm that GMS is a unique edge‐site‐free and topological‐defect‐rich mesoporous carbon material. GMS is ultra‐stable in Li—O_2_ batteries; exhibiting a high discharge capacity of 6727 mAh g^−1^, a low charge plateau at ≈3.6 V (V versus Li/Li^+^), and better electrochemical stability than other commercial carbon materials. In particular, the proposed organic‐binder‐free GMS‐GO‐film shows an ultra‐long cycle life of up to 307 cycles (>1535 h), and good rate performance in Li—O_2_ batteries without either solid catalysts or redox mediators. The low charge plateau is caused by the formation of easily decomposable Li_2_O_2_ nanosheets on the topological defects. This work illustrates the significant enhancement of catalytic activity in Li—O_2_ battery cathodes caused by topological defects, and provides a rational design principle of carbon cathodes in advanced metal–gas batteries.

## Conflict of Interest

The authors declare no conflict of interest.

## Author Contributions

W.Y. and H.N. conceived the idea, designed the experiments, wrote the manuscript, and H.N. directed the project. W.Y., R.T., and Z.Z.P. prepared the materials for the GMS synthesis. W.Y. and R.T. performed the TPD tests. W.Y. conducted the battery tests, the SEM tests, the chemical titration tests, the N_2_ adsorption/desorption tests, and the Raman tests. T.Y. and A.A. performed the DFT calculations. Y.N. synthesized the GO dispersion. K.N. and S.N. performed the DEMS tests. Y.Z. and O.T. performed the TEM tests. K.I. and M.K. performed the mathematical calculations. D.T. and E.S. performed the theoretical calculation of Raman spectra. H.T. drew the schematic of GMS. All authors discussed and analyzed the data.

## Supporting information

Supporting Information

## Data Availability

The data that support the findings of this study are available from the corresponding author upon reasonable request.
