# Supplementary material for: Edge‐Site‐Free and Topological‐Defect‐Rich Carbon Cathode for High‐Performance Lithium‐Oxygen Batteries
Source: Adv Sci (Weinh). 2023 Apr 7;10(16):2300268. doi: 10.1002/advs.202300268 (PMC10238210; doi:10.1002/advs.202300268)
Supplement: Supplementary file 1 — Supporting Information [file ADVS-10-2300268-s001.pdf]

Supporting Information

Edge-Site-Free and Topological-Defect-Rich Carbon Cathode for High-Performance Lithium-Oxygen Batteries

*Wei Yu, Takeharu Yoshii, Alex Aziz, Rui Tang, Zheng-Ze Pan, Kazutoshi Inoue, Motoko Kotani, Hideki Tanaka, Eva Scholtzová, Daniel Tunega, Yuta Nishina, Kiho Nishioka, Shuji Nakanishi, Yi Zhou, Osamu Terasaki and Hirotomo Nishihara**

Experimental Section

Material preparation.

The GMS powder was prepared based on our previous method by using spherical Al_2O_3 nanoparticles (SBa-200, Sasol) as a template.[1] Carbon coating was performed by CVD at 900 °C with a temperature increase rate of 10 °C/min from a room temperature using methane as a carbon source. Depending on the scale of CVD, the CVD period was adjusted to achieve the average graphene stacking layer of 1.1. Then, the template was removed by HF washing to obtain CMS. The CMS was then annealed at 1800 °C with a temperature increase rate of 10 °C/min from a room temperature for 1 h under reduced pressure (Ar atmosphere, 10 Pa) to synthesize GMS.

Seven types of carbon powders, multi-walled carbon nanotubes (CNT, Beijing DK nano technology Co.LTD), carbon black (CB, acetylene, Alfa Aesar), ketjen black (KB, EC-

300J, Lion specialty chemicals Co., Ltd), reduced graphene oxide (rGO, Sigma-Aldrich), activated carbon (AC, MSC-30, Kansai Coke and Chemicals Co. Ltd.), CMS and GMS were used in this work. In the preparation of carbon cathode, carbon powder and polyvinylidene fluoride binder (PVDF, KF polymer L#1120, Kureha Co. Ltd.) were first mixed in N-methyl-2-pyrrolidone (NMP, TCI), and then the mixture was spread onto carbon paper (CP, Toray H060) to form X-CP cathode (X represents the abbreviation of the sample). The loading weight of the carbon sample was about 0.7 mg cm^{-2}. To prepare the free-standing GMS-based film, graphene oxide (GO, 1 wt%, NiSiNa materials) dispersion and GMS powder were dispersed in 50 mL of NMP solvent. The suspension of GMS and GO was formed after ultrasonic irradiation for 1 h and filtered under vacuum using an alumina-based membrane (Whatman anodic inorganic membranes, ϕ 47 mm). Finally, after filtration and the subsequent heating process (40 ^{o}C), a free-standing GMS-GO-film with an areal density of 2.7 mg $cm^{-2}{}_{GMS}$ and a thickness of 80 μm was fabricated. All carbon cathodes were dried under vacuum at 110 °C for 24 h.

Battery assembly and electrochemical tests.

2032-type coin-cells with 17 holes (ϕ 1 mm) in a cathode cap were used for the assembly of Li-O_2 batteries in this work. The electrolyte used in Li-O_2 batteries was 0.5 M lithium bis(trifluoromethanesulfonyl)imide (LiTFSI, Sigma-Aldrich) in tetraethylene glycol dimethyl ether (TEGDME, Sigma-Aldrich). The TEGDME solvent was stored with 4 Å molecular sieves for more than 2 weeks to remove water content and the Li salts were carefully dried under vacuum at 110 ^{o}C for 24 h. The TEGDME-based electrolyte infiltrated in a glass fiber separator was about 100 μL for each Li-O_2 battery. A piece of Li metal (ϕ 16 mm) was used as an anode, which was separated from the

carbon cathode by a piece of glass fiber separator (GF/D, Whatman). In addition, all components of the cell, including the springs, spacers, a porous current collector (nickel foam), and a GF separator were dried under vacuum at 110 °C for 24 h before being transferred into a glovebox. All coin cells were assembled and disassembled in a highly purified Ar-filled glovebox. The coin cells were settled in a homemade test device filled with high-purity O_2 (99.995%) and allowed to stand for 4 h before any tests. CV tests of the two-electrode coin-cells were performed on a VMP3 electrochemical workstation (Bio-Logic Science Instruments) with a voltage range between 2.0 and 4.6 V (vs Li/Li^+) at a scan rate of 0.1 mV s^{-1}. All galvanostatic discharge-charge tests were performed using an HJ-SD 8 (HOKUTO DENKO) at room temperature with a cutoff voltage of 2.0-4.6 V (vs. Li/Li^+). GITT was performed with a Li-O_2 battery at a current density of 0.2 mA cm^{-2} and a limited capacity of 1.0 mAh cm^{-2} with a potential range of 2.3 to 4.6 V (vs. Li/Li^+). During GITT measurements, the batteries after discharging were charged by a short pulse of constant current for 10-min and followed by a 3-hours time interval. This procedure was repeated until the total charging capacity reached 1.0 mAh cm^{-2} or the charge potential reached 4.6 V (vs. Li/Li^+).

Characterizations.

Advanced TPD up to 1800 ^{o}C was used to quantify the edge sites by determining the total gas evolution of carbon materials.[2] X-ray photoelectron spectroscopy (XPS) measurements towards CMS and GMS powders were carried out by a PHI Quantera II spectrometer (Ulvac-Phi) with a monochromatic Al Kα excitation source. Thermogravimetry (TG) analysis was performed in a TG analyzer (TGA-50, Shimadzu) under an airflow with an upper temperature of 900 ^{o}C. N_2 adsorption/desorption tests were performed at −196 ^{o}C using a volumetric sorption analyzer (BEL Japan,

BELSORP MAX). The surface area of carbons was calculated by using the BET method. Total pore volume was determined from the N_2 adsorption amount at P/P_0=0.96. The mesopore size distribution was calculated by using the Barrett-Joyner-Halenda (BJH) method applied to the N_2 adsorption isotherm. Raman spectra were measured using a Raman spectroscopy system (LabRAM HR800, HORIBA Jobin Yvon). An excitation wavelength of 532 nm in a 180° reflection mode with a wavenumber range of 100 to 3500 cm^{-1} was used. To prepare TEM samples, the GMS powder was first ultrasonically dispersed in ethanol and then the dispersed droplets were deposited on the Cu grid cover with lacey carbon. The TEM images were acquired on a JEM-ARM300F GRAND ARM (JEOL) equipped with double Cs correctors with an acceleration voltage of 80 kV. The micromorphology of the cathodes before and after electrochemical tests was characterized using a scanning electron microscope (SEM, S-4800, Hitachi). Secondary electrons were detected as the signal by the upper detector placed above the objective lens. SEM images were taken at the electron impact energy of 5.0 keV. The carbon cathodes were washed with 1,2-dimethoxyethane (DME) after electrochemical tests to remove residual electrolytes and Li salts. XRD was applied to characterize the crystalline phase of the products on the carbon cathodes, operated on a MiniFlex600 diffractometer (Rigaku) at 40 kV and 40 mA. All cathodes for XRD characterization were sealed in the air-tight device covered with a piece of Kapton film. Quantitative analysis of the formation and decomposition of Li_2O_2 in GMS-CP cathodes was performed by chemical titration measurement. 5 mL of $TiOSO_4$ (Aldrich, ~2 wt% in sulfuric acid) solvent was added to an air-tight glass bottle containing a GMS-CP cathode after discharge or charge. Absorbance was measured at 409 nm using an ultraviolet-visible spectrophotometer (UV-vis, JASCO, V-670). The amount of Li_2O_2 in

the GMS-CP cathode was calculated using an absorbance-weight calibration curve. To characterize the discharge-charge mechanism of GMS-based Li-O_2 batteries, both conventional $^{16}O_2$ and isotope $^{16}O_2/^{18}O_2$ DEMS were conducted with a quadrupole mass spectrometer (PrismaPlus QMG220, Pfeiffer Vacuum, Germany).[3] During the DEMS analysis, a gas flow of approximately 1 mL/min was introduced into the mass spectrometer. The amount of gas evolved in each test was quantified based on the signal from the instrument and prior calibration with a gas sample of known concentration.

Theoretical calculations.

Mathematical calculation.

Spherical Voronoi analysis was applied to estimate the total length of graphene boundaries on the Al_2O_3 nanoparticle. An Al_2O_3 nanoparticle was idealized by a sphere with randomly distributed base points. The sphere diameter was set D=9.0 nm to fit the average size of Al_2O_3 nanoparticles where the base points correspond to the active sites for graphene growth, and each pair of base points was separated by no less than 1.0 nm. Then, 1000 configurations are generated for each N=2-130, and the Voronoi tessellation was applied to the set of N base points on the spherical surface. The average of the total length L of Voronoi edges is presented in Figure 3 with the standard deviation. We note that the spherical Voronoi edge is a part of the intersection between the sphere and the perpendicular bisector of a pair of base points, where is an arc of the great circle of the diameter D. Therefore, L coincides with the arc length of the great circle for N=2. For N=3 as shown in the inset Figure 3, Voronoi vertices are the circumcenter of the spherical triangle and its antipodal point, and hence L is 1.5 times longer than the case in N=2.

Modeling.

Carbon atoms (2946 atoms) were randomly placed on the surface of hypothetical spherical alumina (4.66 nm in diameter) using a Monte Carlo method. Then, a quench molecular dynamics (QMD) simulation was performed to make carbon atoms react with each other on the surface of spherical alumina (homemade simulation code). The reaction state summation potential[4] was applied for the interaction between carbon atoms and the Lennard-Jones (LJ) potential for the interaction between carbon atoms and the alumina surface. The hypothetical alumina surface structure was assumed to be a spherical shell with one layer of atoms and uniformly distributed atoms. To prevent the release of carbon atoms from the alumina surface during the QMD at high temperature, the LJ parameters for the interaction between solid atoms and carbon atoms were set to ε_{sf}/k = 70000 K (k: Boltzmann constant) and σ_{sf} = 0.34 nm. The initial temperature was set to 10000 K and quenched to 298 K by the velocity scaling method (quench rate: 13 K/ps). The equation of motion was integrated by the velocity Verlet algorithm with a time step of 0.05 fs and a total QMD time of 750 ps. For the structure obtained by QMD, the spherical alumina was removed and all carbon atoms with a coordination number of one were deleted. Then, NVT-MD simulation was performed (LAMMPS software[5]) using the resulting spherical carbon structure (2723 atoms) and structural relaxation was performed at 298 K. The AIREBO potential[6] was used for the carbon interatomic potential. The equations of motion were integrated by the velocity Verlet method, with a time step of 0.05 fs and a total MD time of 15 ps. The temperature was controlled by the Nose-Hoover method.

Raman spectra.

To obtain the theoretical characteristic bands in the Raman spectra of the structural

models of pristine graphene with only hexagonal rings and graphene with topological defects (5,7-membered rings), the *Ab initio* DFT method was employed using Vienna *Ab Initio* Simulation Package (VASP).[7] The exchange-correlation energy was expressed in the frame of the generalized gradient approximation (GGA) using the functional proposed by Perdew, Burke and Ernzerhof (PBE).[8] The Kohn-Sham equations were solved in a plane-wave (PW) basis set with an energy cut-off of 500 eV at the *gamma* point. Electron-ion interactions were described using the projector-augmented-wave (PAW) method.[9] Relaxation criteria were 10^{-5} eV/atom for the total energy change and 0.005 eV/ Å for the maximum allowable forces acting on each atom. Dispersion corrections were included in the D3 scheme.[10] The normal modes of the vibrations were calculated within the fixed optimized cells using a finite difference method, and the normal mode analysis was performed in the harmonic approximation framework. The Hessian was constructed from the single-point energy calculations for the 6n structures generated from the optimized structures by shifting each of the n atoms in the cell in a positive and negative sense along the Cartesian directions *x*, *y*, and *z*.[11]

DFT calculation.

To obtain a free energy diagram of the ORR/OER process on graphene with only hexagonal rings and graphene with topological defects (5,7-membered rings), DFT calculations were performed using the plane-wave self-consistent field (PWscf) code in the Quantum ESPRESSO package.[12] All calculations were carried out using the functional proposed by PBE[8] and ultrasoft pseudopotentials[13] with plane-wave and charge-density cutoffs of 25 and 225 Ry, respectively. Relaxation criteria were 1.0×10^{-4} Ry for the total energy change and 1.0×10^{-3} Ry/Bohr for the maximum allowable forces acting on each atom. Two types of slabs consisting of a single layer with 64

carbon atoms were employed as the model substrates: (i) a (5 × 2) graphene supercell with only hexagonal rings and (ii) a (5 × 2) graphene supercell with topological defects (5,7-membered rings). A vacuum gap spacing of 20 Å was used to prevent self-interaction. A Monkhorst–Pack *k*-point samplings of (2 × 2 × 1) were used, and an electron smearing was employed with a width of $k_BT = 0.01$ Ry using the Methfessel–Paxton smearing method to aid *k*-point convergence. The adsorption energy (E_{ads}) was defined as follows:

$E_{ads} = E_{Adsorbate/Substrate} - (E_{Substrate} + E_{Adsorbate})$ (1)

where $E_{Substrate}$ is the total energy of the substrate, $E_{Adsorbate}$ is the total energy of the free adsorbate, and $E_{Adsorbate/Substrate}$ is the energy of the substrate together with the adsorbate. Thus, negative values of E_{ads} correspond to an exothermic adsorption process. In calculating the adsorption energies of O_2 and Li on the substrate, the DFT energies of O_2(g) and Li(s) were used as $E_{Adsorbate}$, respectively. The reaction free energy (ΔG) was calculated as follows:

$\Delta G = E_{Inter} + \Delta N\mathrm{O_2} \cdot \mu\mathrm{O_2} + \Delta N\mathrm{Li} \cdot (\mu\mathrm{Li} - eU) - E_{Final}$ (2)

where E_{Inter} and E_{Final} are the DFT total energy of the intermediate and final step, $\Delta N\mathrm{O_2}$ and $\Delta N\mathrm{Li}$ are the numbers of O_2 and Li, and $\mu\mathrm{O_2}$ and $\mu\mathrm{Li}$ are the chemical potentials of O_2(g) and Li(s), respectively. Zero-point energy (ZPE) and entropic corrections were applied based on the reported values.[14] The following software was used to create the input files: *Winmostar V10, X-Ability Co. Ltd., Tokyo, Japan, 2020*. All optimized structures, electron density contours, and charge difference plots were visualized using the program VESTA.[15] The discharge potential and charge potentials are the highest discharge potential and lowest charge potential at which the free energy goes downhill in each ORR/OER step.

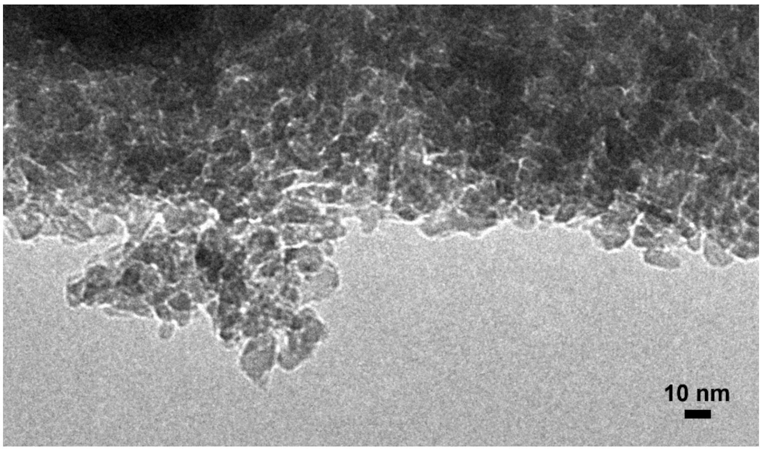

Figure S1 TEM image of Al_2O_3 nanoparticles.

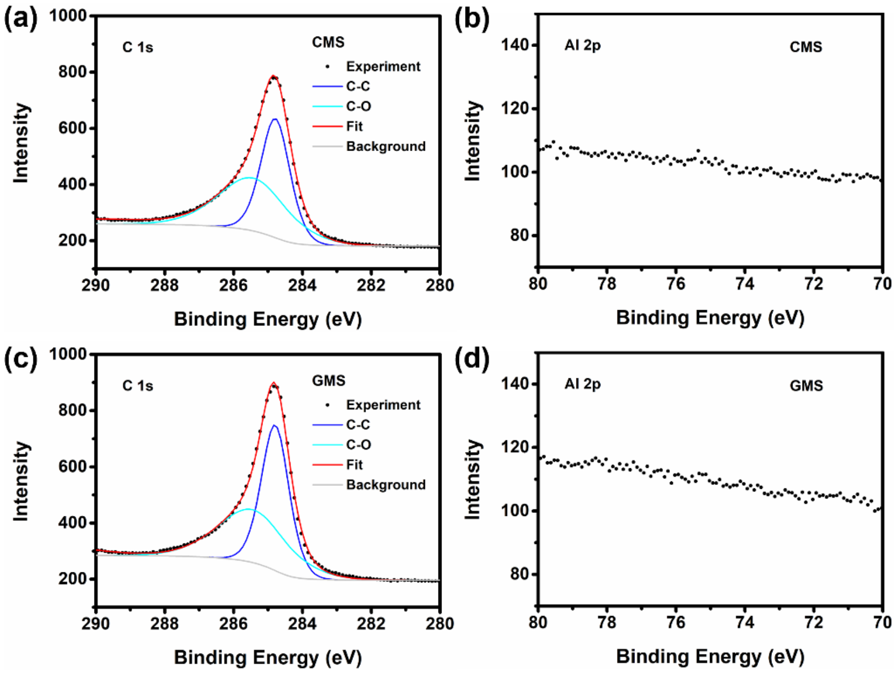

Figure S2 XPS spectra of (a, b) CMS and (c, d) GMS powders. (a, c) C 1s and (b, d) Al 2p.

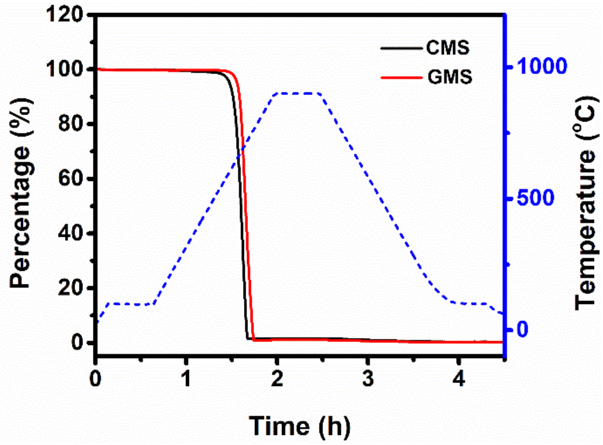

Figure S3 TG curves measured in the air for CMS and GMS powders.

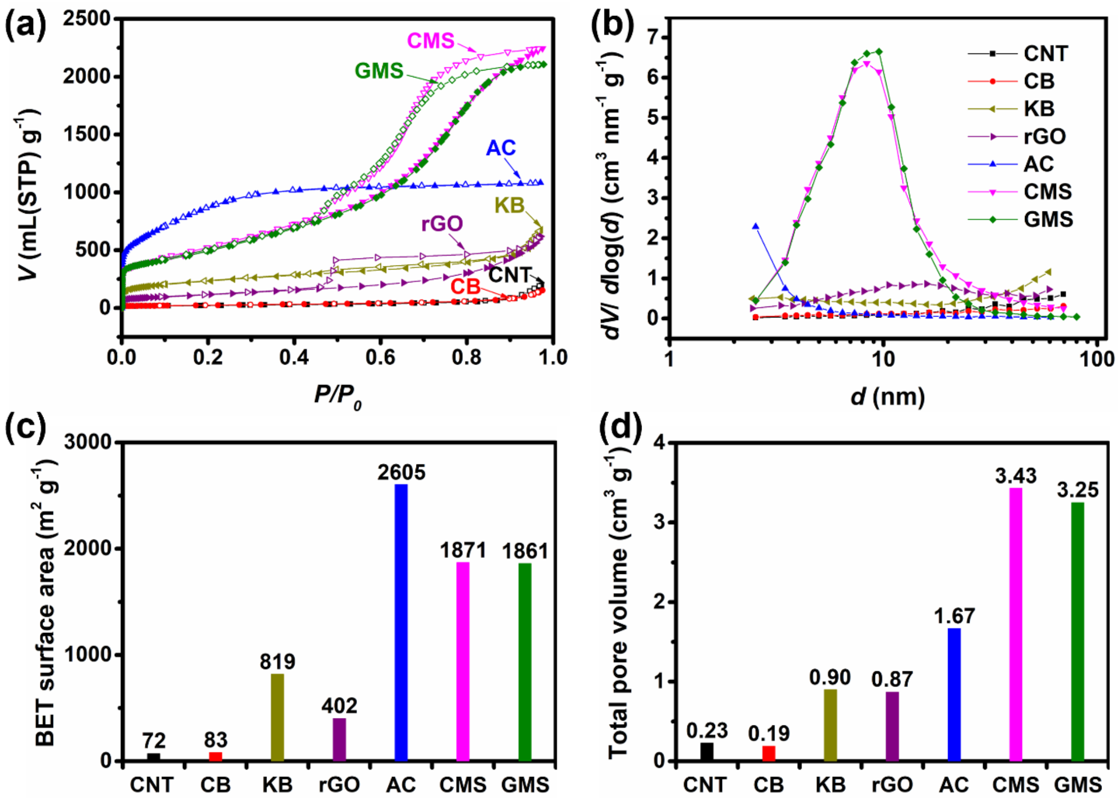

Figure S4 (a) N_2 adsorption/desorption isotherms of GMS and other six reference carbon powders. (b) Pore size distributions calculated using the Barrett–Joyner–Halenda (BJH) method. (c) BET surface areas. (d) Total pore volumes calculated from the N_2 adsorption amount at $P/P_0 = 0.96$.

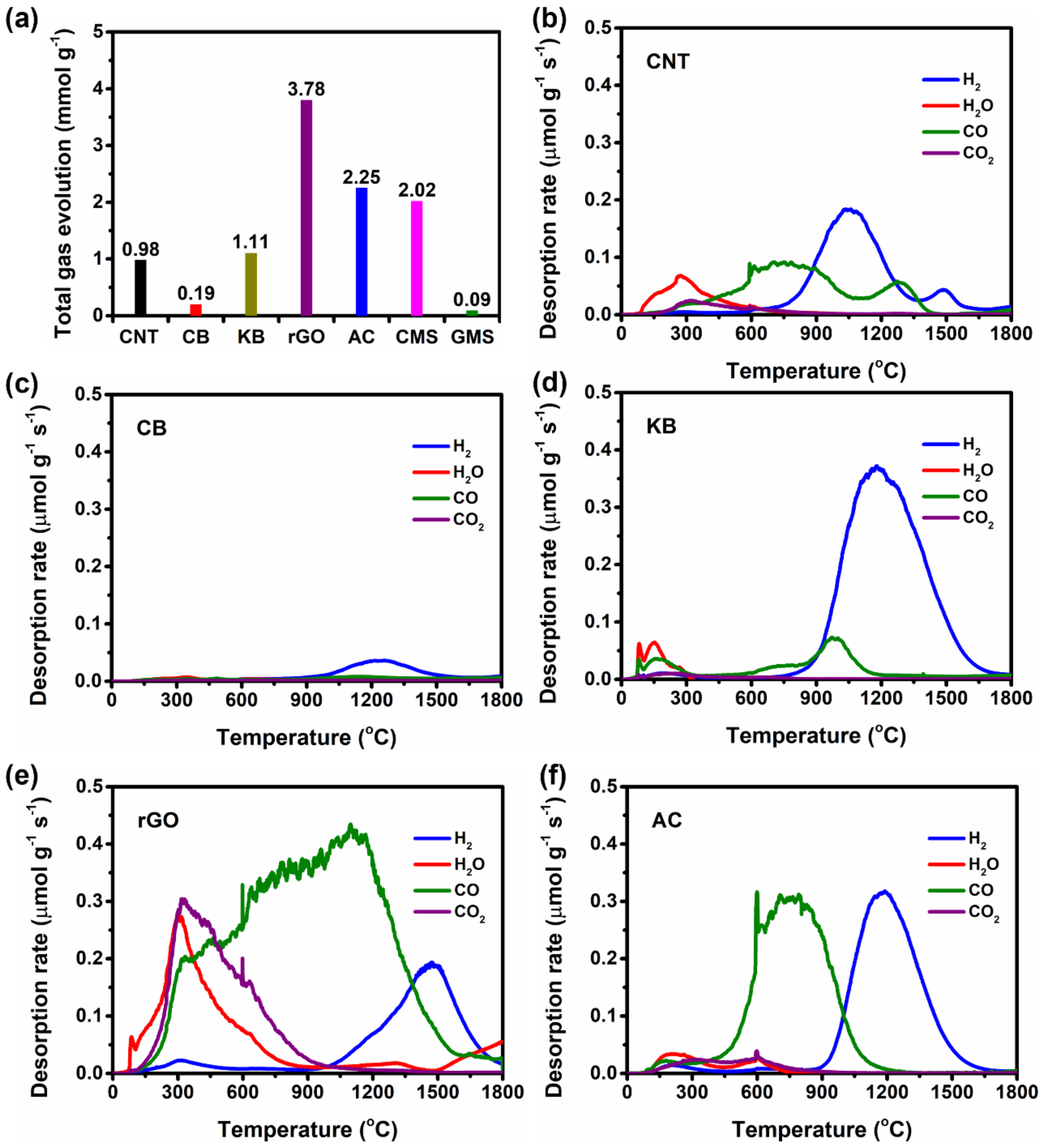

Figure S5 (a) Total gas evolution during the TPD analysis for GMS and reference carbons. The total gas evolution reflects the amount of carbon edge sites. Gas evolution patterns of H_2, CO, CO_2, and H_2O during the TPD measurements for (b) CNT, (c) CB, (d) KB, (e) rGO and (f) AC.

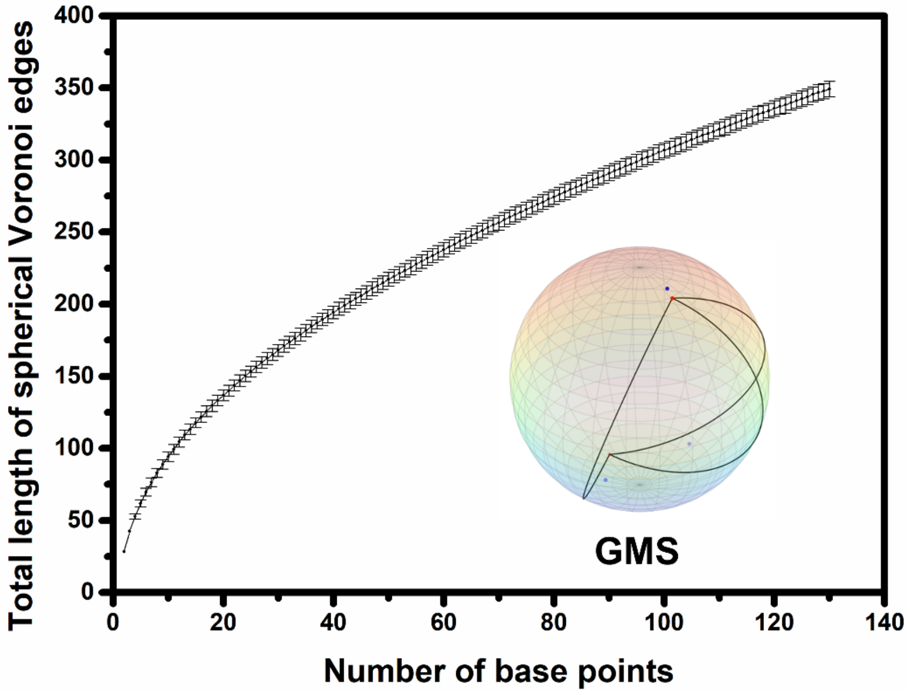

Figure S6 The average total length of the Voronoi edges for each number of base points corresponding to active sites on the sphere of diameter D=9.0 nm with the standard deviation. Insert: the schematic corresponding to GMS with N=3 base points (blue dots), Voronoi vertices (red dots), and Voronoi edges (gray curves).

Supplementary Note 1

Calculation of total length of graphene boundaries in GMS

Al_2O_3 nanoparticles (SBa-200, Sasol Chemical Co., γ-alumina) with an average particle size of 9.0 nm were used as a template to synthesize CMS and GMS. After CVD, the carbon-loading amount on a single Al_2O_3 nanoparticle is around 2.01×10^{-19} g. According to the gas emission amount of CMS from the TPD test (Figure 1a), the total number of edge-sites was estimated as 3046 μmol g^{-1} based on the equation of $2\times H_2$ (1097 μmol g^{-1}) + H_2O (> 400 °C, 90 μmol g^{-1}) + CO (659 μmol g^{-1}) + CO_2 (103 μmol g^{-1}).[16] Therefore, the total number of edge sites (n_{edge}) corresponding to a single Al_2O_3

nanoparticle is 368. Also, the total length of boundary (assuming 50%/50% armchair/zig-zag edges) estimated from TPD results (n_{edge}) is 42 nm, which corresponds to the basal point $\boldsymbol{N}$ = 3 case in the mathematical simulation and proves the existence of domain boundaries in GMS (Figure S6).

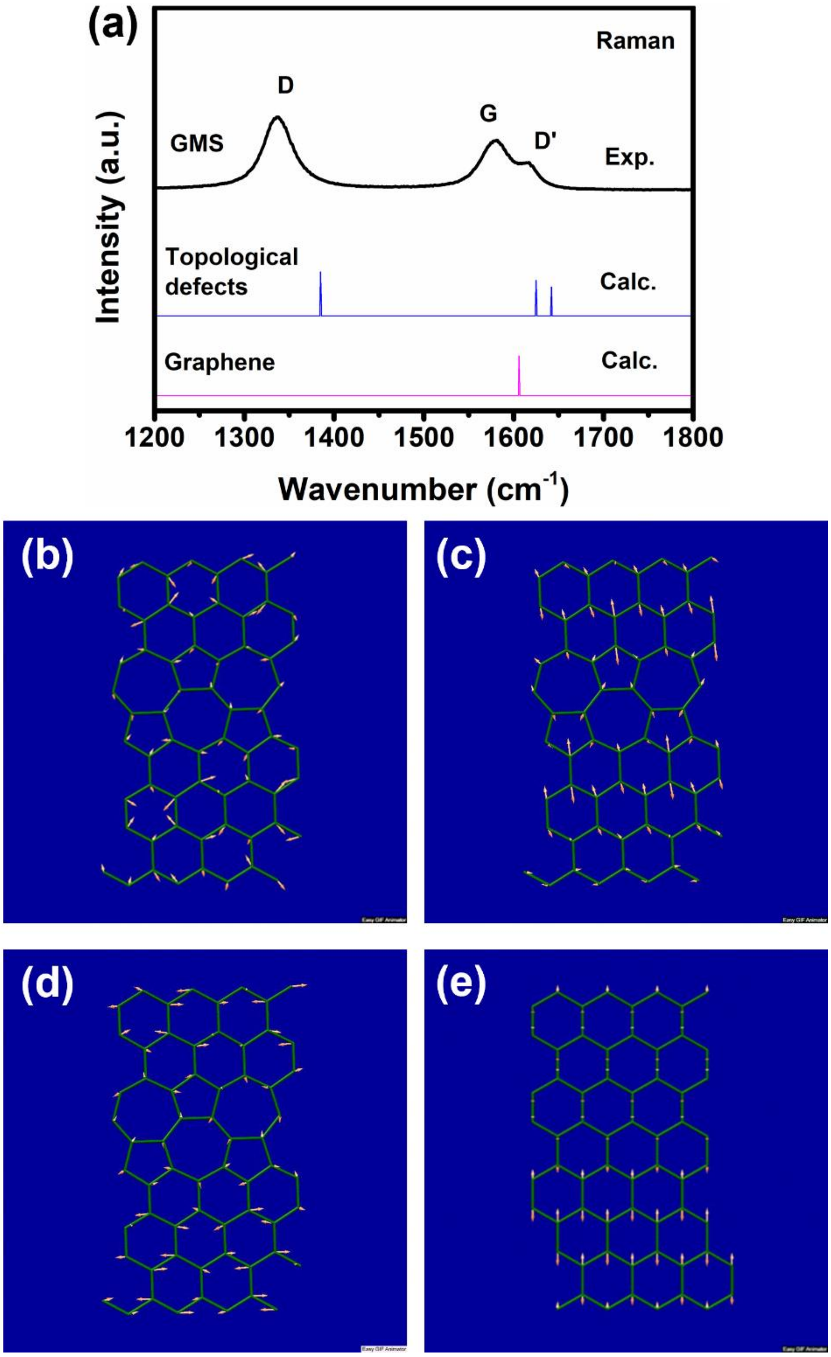

Figure S7 (a) Raman spectra of GMS, together with theoretical Raman bands of planar graphene and graphene including topological defects. Calculated vibration of (b) D-band (1385 cm^{-1}), (c) G-band (1625 cm^{-1}), (d) D'-band (1642 cm^{-1}) in GMS with 5,7-carbon rings and (e) G-band (1606 cm^{-1}) in perfect graphene.

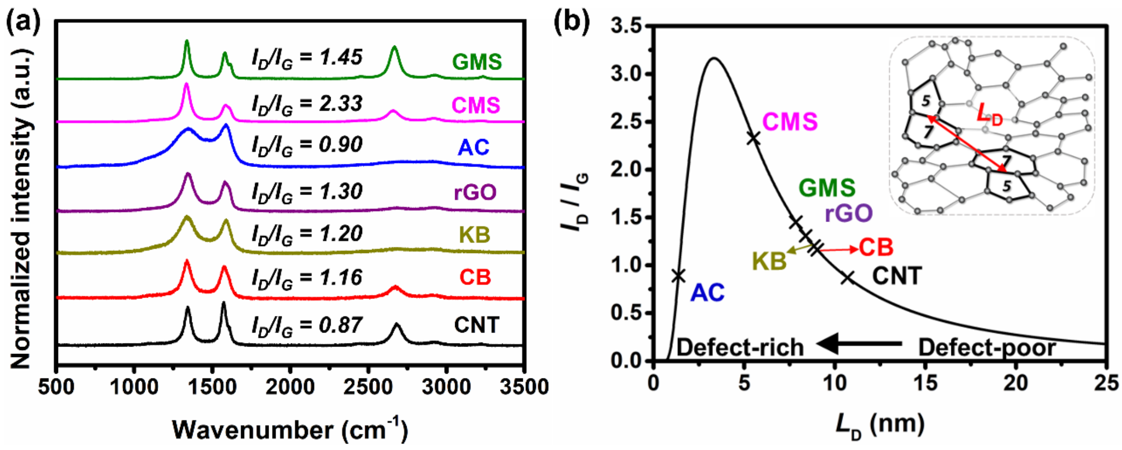

Figure S8 (a) Raman spectra of CMS, GMS, and reference carbons. The intensity ratio of D-band (1337 cm^{-1}) and G-band (1580 cm^{-1}), I_D/I_G ratio, is described for each sample. (b) The correlation between I_D/I_G ratio and the mean distance between defects (L_D) and the plots of different carbons in the fitted line. The fitted line is plotted using parameters of r_S = 1 nm, r_A = 3.1 nm, and CA = 4.2 for the formula (S1). The inset illustrates the mean distance between topological defects (L_D) for GMS.

Note S2

Quantifying defects by Raman spectroscopy

Raman spectroscopy was used to quantify the defects in carbon material.[17] As shown in Figure S7a, the G band around 1580 cm^{-1} corresponds to a primary in-plane vibration mode. And the D-band around 1340 cm^{-1} indicates the breathing modes of carbon rings

and is the characteristics of defects.[18] As shown in Figure S8b, an empirical formula has been proposed to correlate the intensity ratio of D band and G band (I_D/I_G ratio) and the mean distance between defects (L_D)[19]:

$$\frac{I_D}{I_G} = C_A \frac{(r_A^2 - r_S^2)}{(r_A^2 - 2r_S^2)} \left[e^{-\frac{\pi r_S^2}{L_D^2}} - e^{-\frac{\pi (r_A^2 - r_S^2)}{L_D^2}} \right] \quad \text{(S1)}$$

where the radii of the "structural disordered" area, r_S (1 nm), and the radii of the "activated" area, r_A (3.1 nm), are used in our calculation, respectively.[19] Due to the use of the same green line excitation (532 nm), the same value of C_A (4.2), the maximum possible value of the I_D/I_G ratio, was also used in our calculation.[20] We plotted different carbon samples on the fitted line by considering the I_D/I_G ratio and estimated domain size.

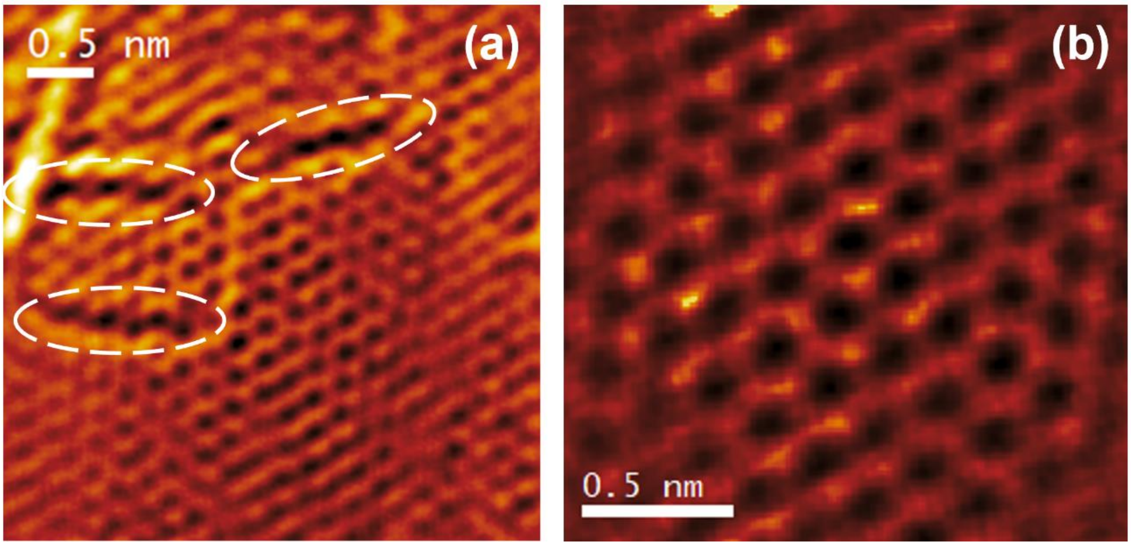

Figure S9 TEM images of GMS showing (a) grain boundaries and (b) 6-membered rings on the basal plane without defects.

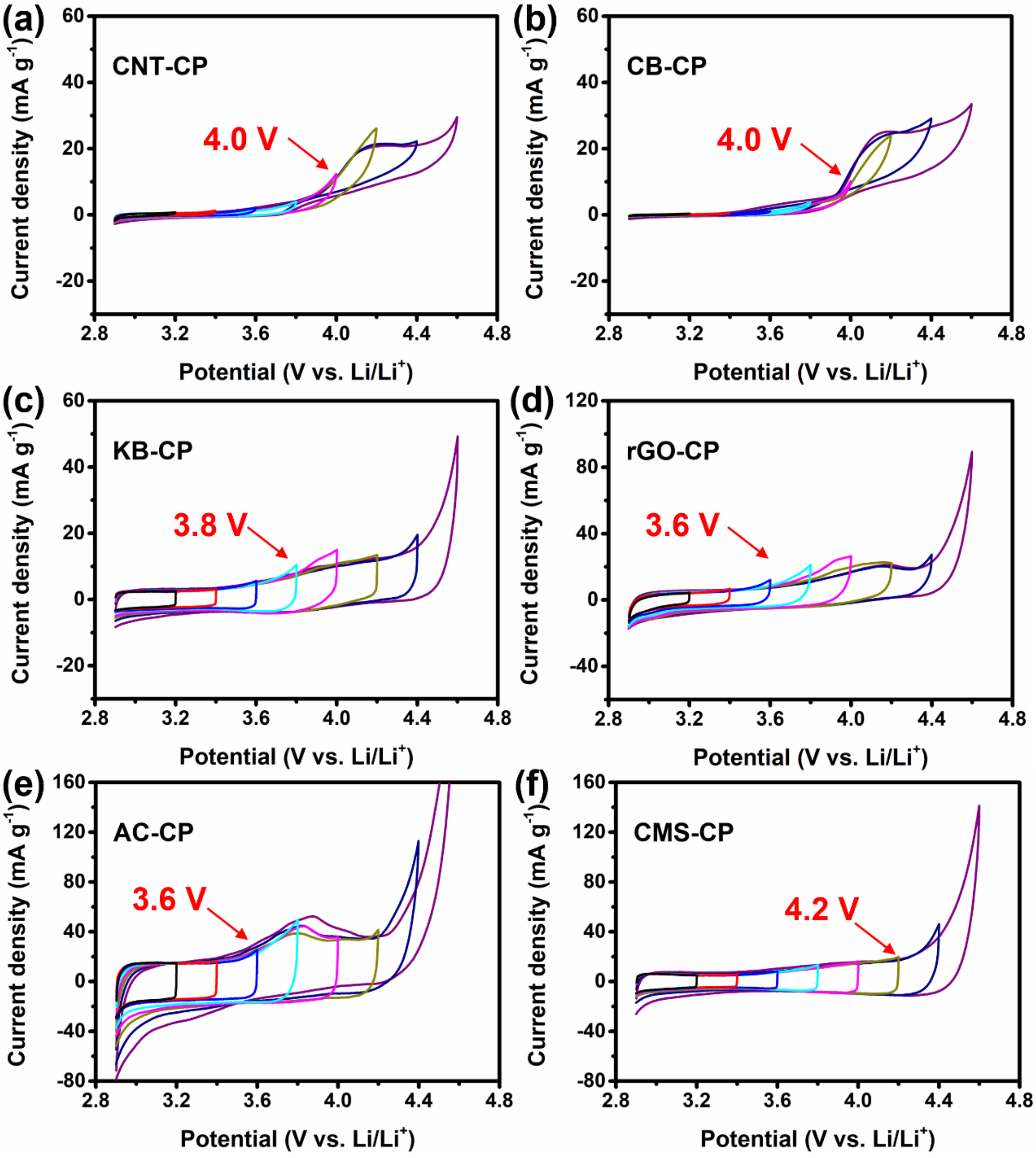

Figure S10 Cyclic voltammograms towards positive potential at 0.1 mV s^{-1} for (a) CNT-CP, (b) CB-CP, (c) KB-CP, (d) rGO-CP, (e) AC-CP, and (f) CMS-CP carbon cathodes. The upper-limit potential of these Li-O_2 batteries with an O_2-saturated electrolyte is increased stepwise from 3.2 V to 4.6 V (vs. Li/Li^+) at 25 ^oC. The onset potentials of anodic oxidation are described.

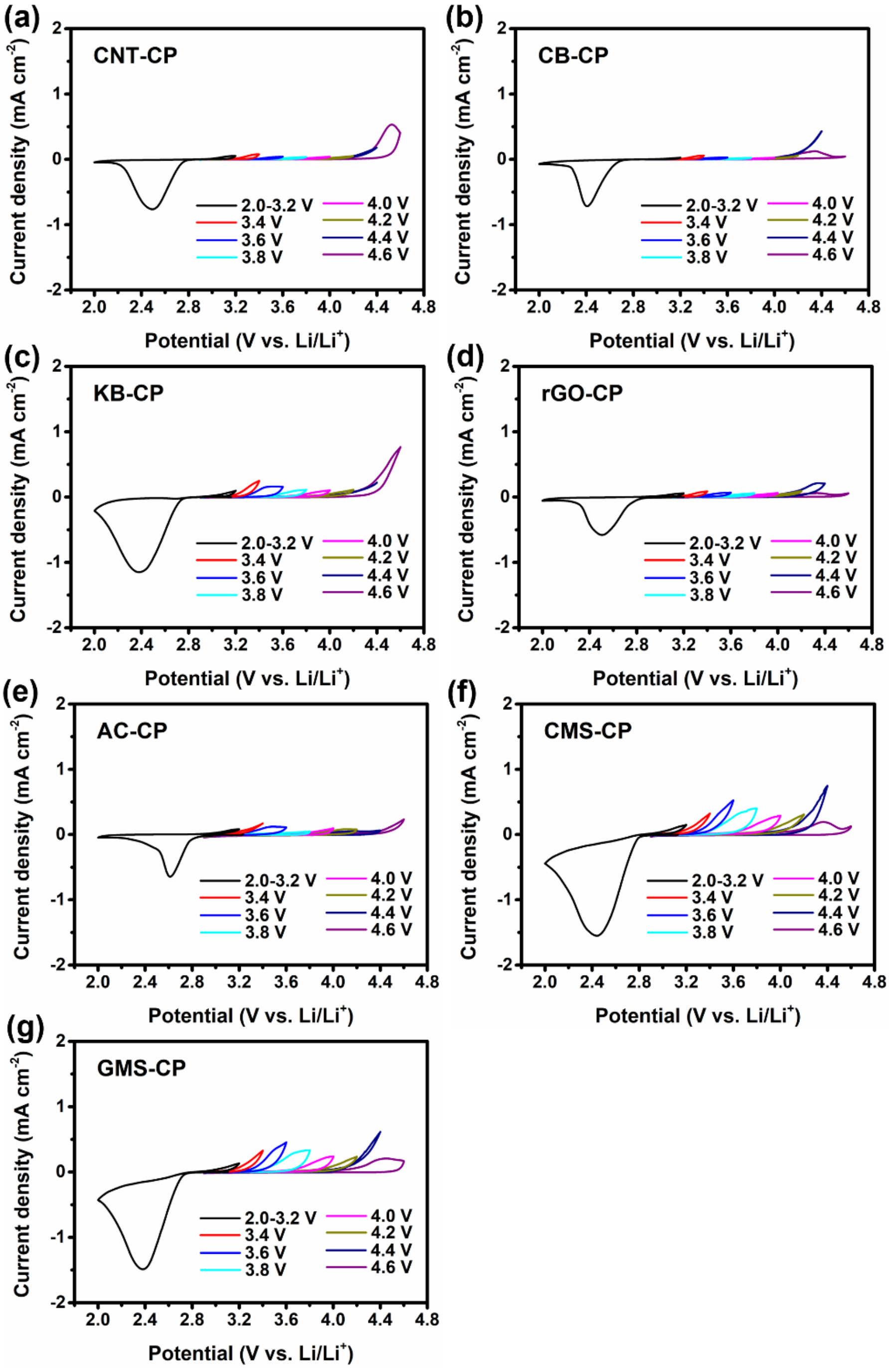

Figure S11 Cyclic voltammograms in a wider potential range with 0.1 mV s^{-1} for (a) CNT-CP, (b) CB-CP, (c) KB-CP, (d) rGO-CP, (e) AC-CP, (f) CMS-CP, and (g) GMS-CP carbon cathodes. The lower limited potential of these Li-O_2 batteries with an O_2-saturated electrolyte is 2.0 V (vs. Li/Li$^+$), and the upper-limit potential is increased stepwise from 3.2 V to 4.6 V (vs. Li/Li$^+$) at 25 °C.

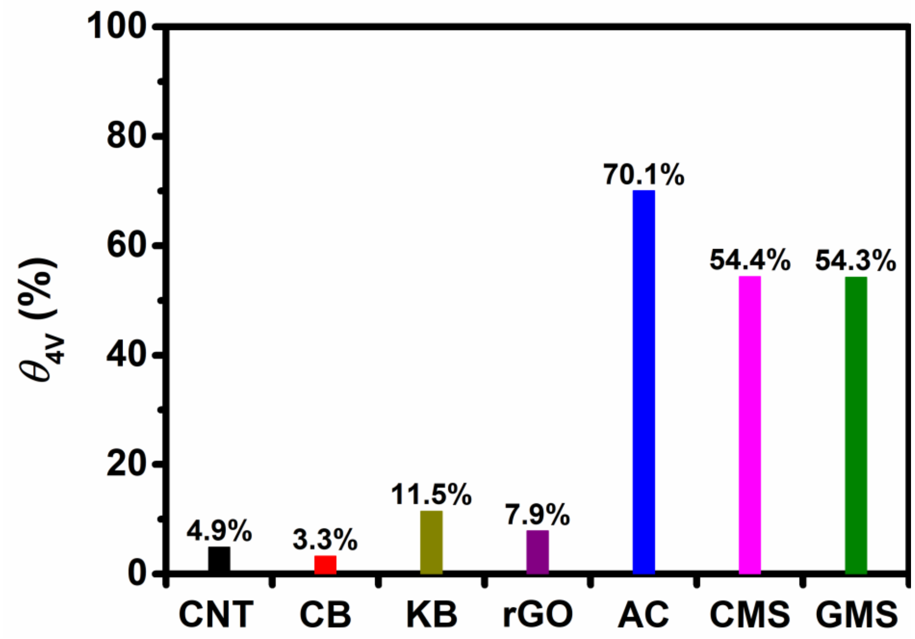

Figure S12 Comparison of the charge-capacity ratio under 4.0 V (θ_{4V}) in the full-discharge-charge tests.

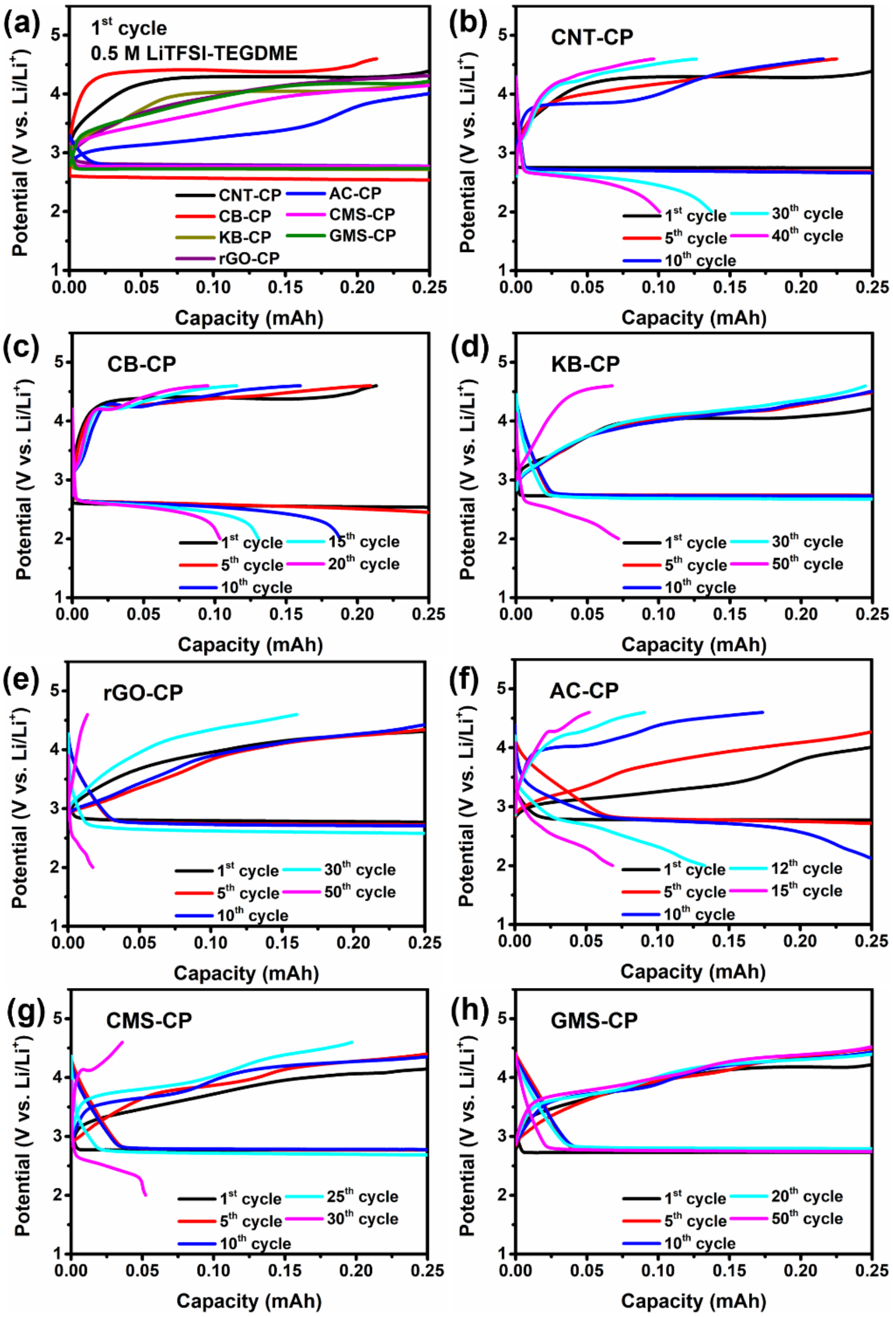

Figure S13 Galvanostatic discharge-charge curves of the Li-O_2 batteries tested with a current of 0.1 mA, a limited capacity of 0.25 mAh and a voltage range from 2.0 to 4.8 V (vs. Li/Li^+). (a) The first cycles of all the samples and (b-h) following cycles for (b) CNT-CP, (c) CB-CP, (d) KB-CP, (e) rGO-CP, (f) AC-CP, (g) CMS-CP and (h) GMS-CP cathodes.

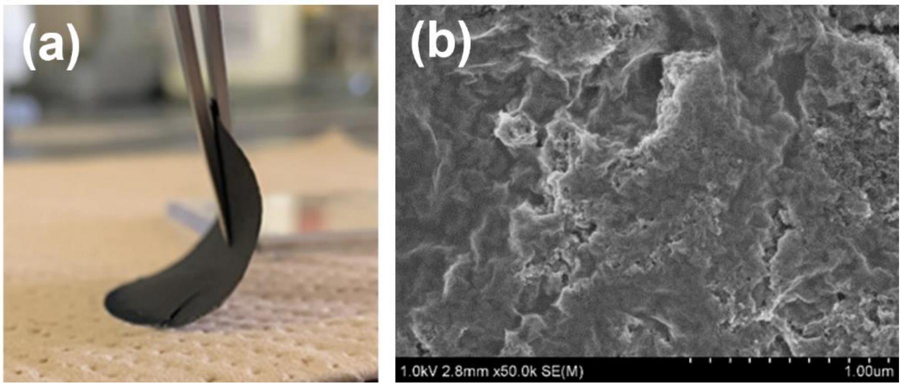

Figure S14 (a) Digital photo and (b) SEM image of GMS-GO-film.

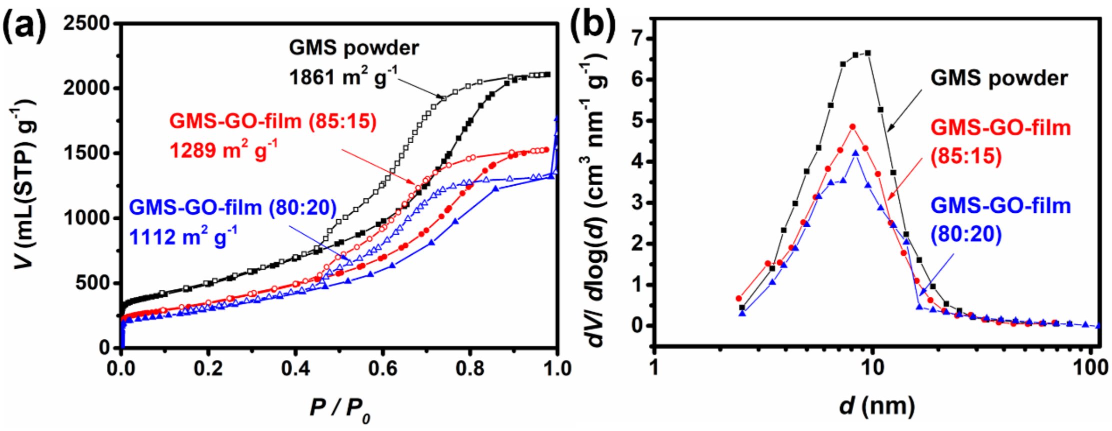

Figure S15 (a) N_2 adsorption/desorption isotherms and (b) pore size distributions. The weight ratio of GMS and GO is described in parentheses.

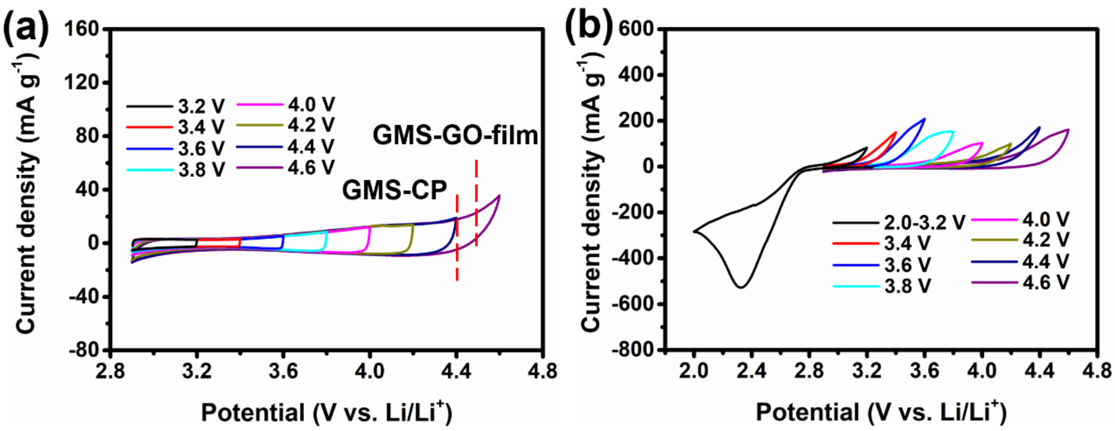

Figure S16 Cyclic voltammograms with (a) only positive-scanning and (b) negative-positive-scanning of the $Li-O_2$ batteries based on GMS-GO-film (85:15) carbon cathodes. The stepwise expanding upper limited potential is from 3.2 V to 4.6 V (vs. Li/Li^+) with a scan rate of 0.1 mV s^{-1}. In Figure S16a, the onset potentials of anodic oxidation are described for GMS-CP and GMS-GO-film.

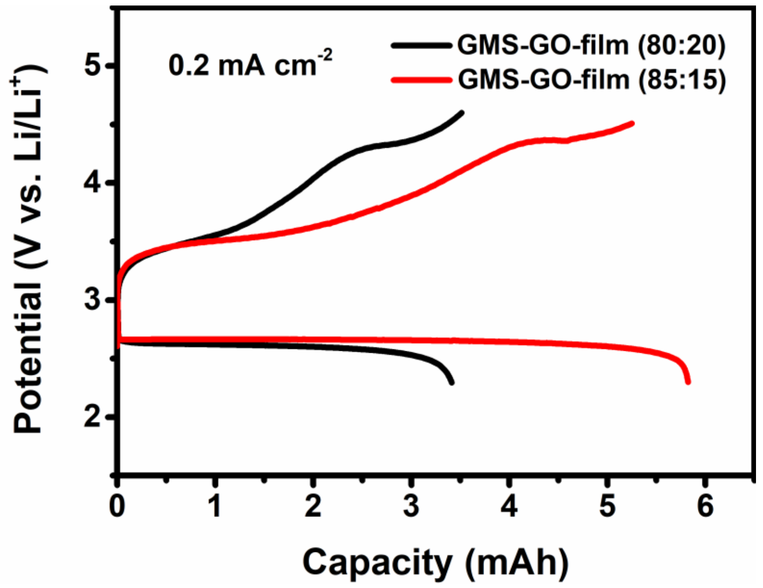

Figure S17 Galvanostatic full-discharge-charge curves of GMS-GO-films based $Li-O_2$ batteries with a voltage range from 2.3 to 4.6 V (vs. Li/Li^+). The weight ratio of GMS and GO is described in parentheses.

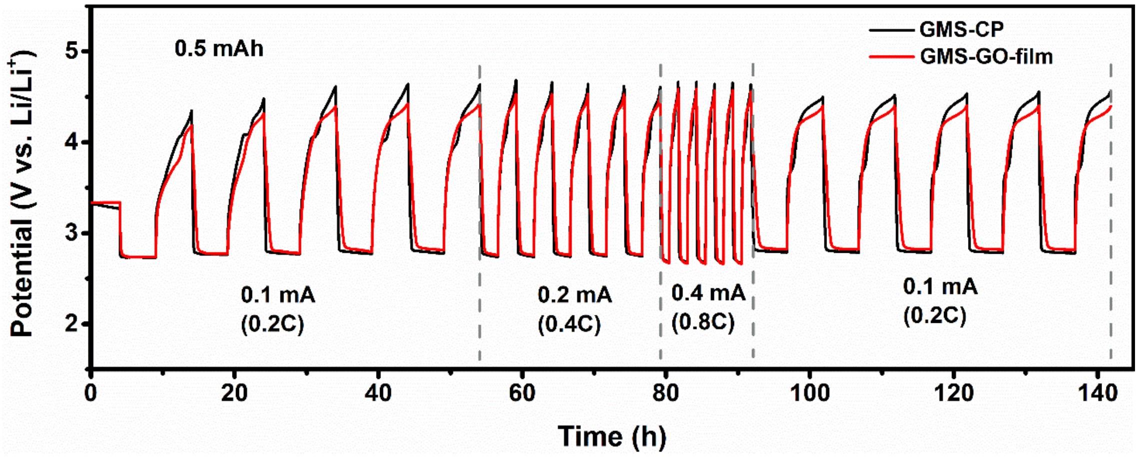

Figure S18 Rate performance of GMS-CP and GMS-GO-film (85:15). Electrochemical measurements were obtained using a coin cell in an O_2-saturated 0.5 M LiTFSI/TEGDME with a limited capacity of 0.5 mAh under different current of 0.1 mA (0.2C), 0.2 mA (0.4C) and 0.4 mA (0.8C).

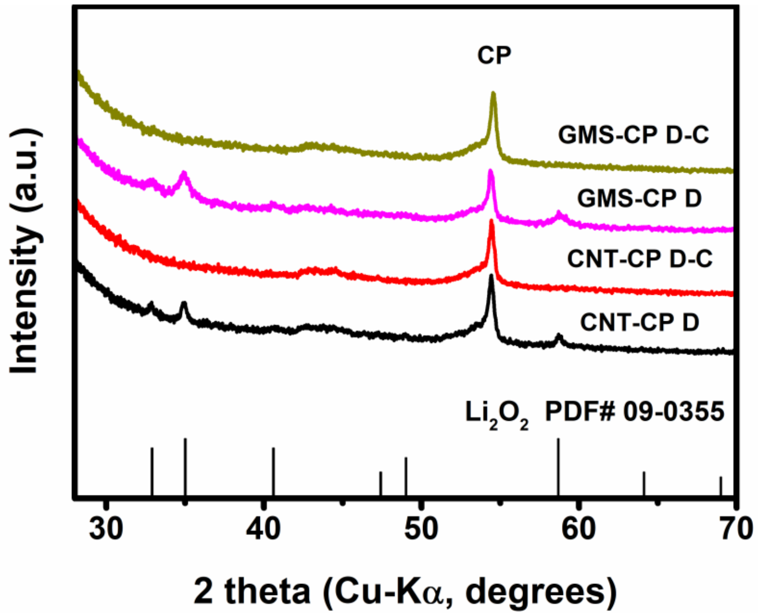

Figure S19 XRD patterns of GMS-CP and CNT-CP after full discharge ("D" after

electrode name) and full charge ("D-C" after electrode name). As a reference, the standard XRD data of Li_2O_2 is shown at the bottom.

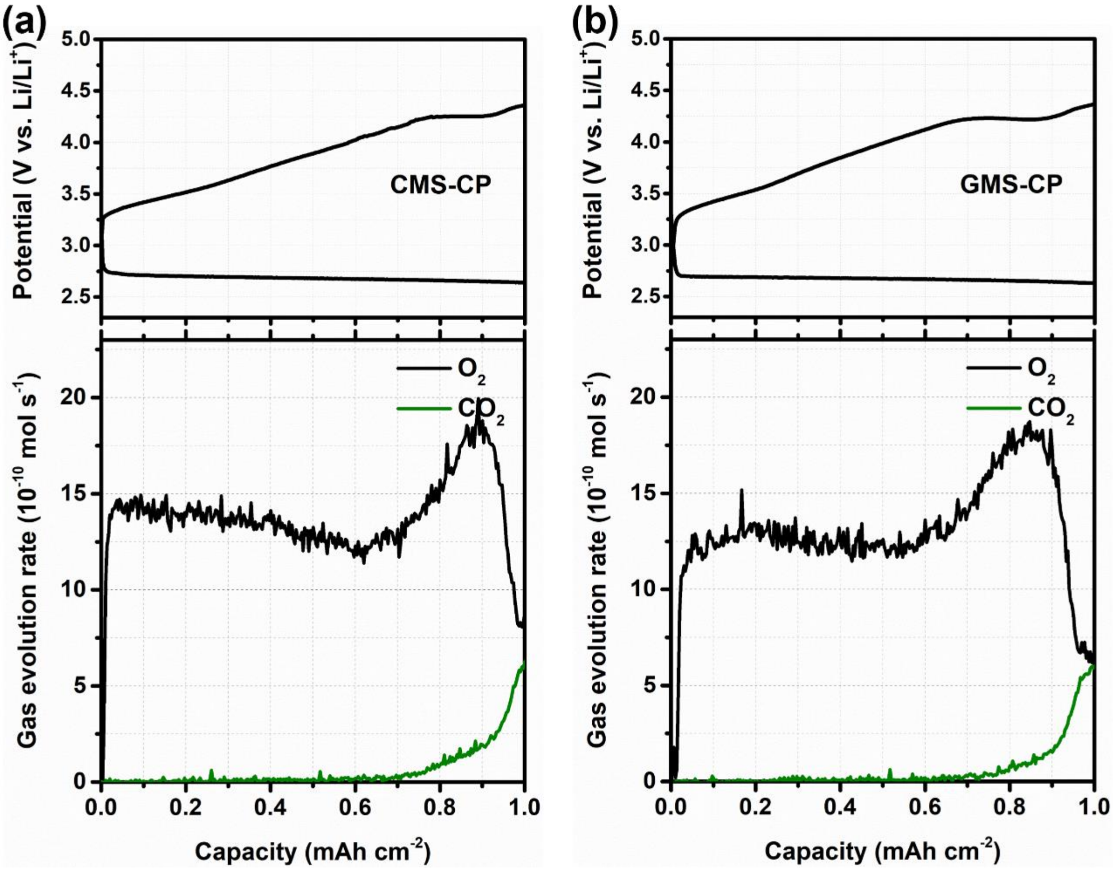

Figure S20 Galvanostatic discharge-charge curves and O_2 evolution rate during the charging process of Li-O_2 batteries in the DEMS test with (a) CMS-CP and (b) GMS-CP under a current density of 0.2 mA cm^{-2} and a limited capacity of 1.0 mAh cm^{-2}.

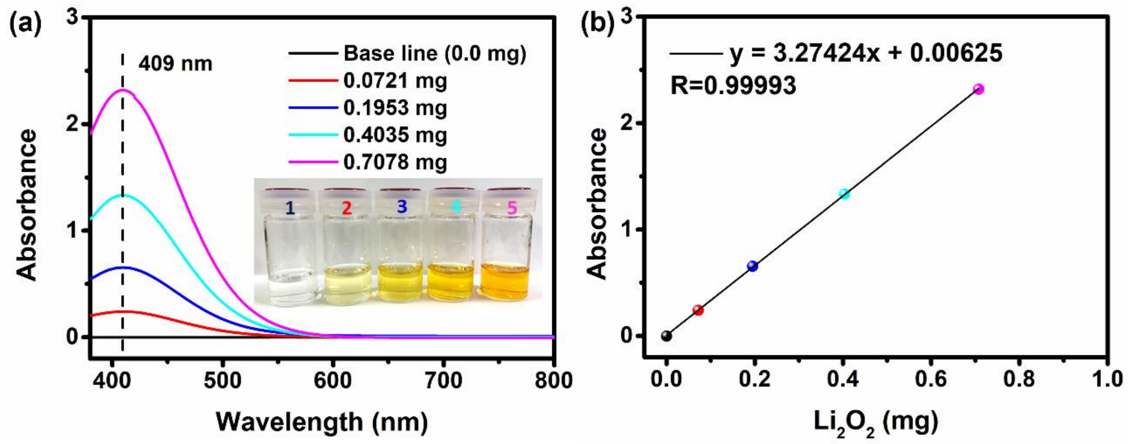

Figure S21 (a) UV-vis spectra of 5 mL $TiOSO_4$ solutions (*aq*) mixed with different quantities of commercial Li_2O_2. An inset of Figure S21a shows digital photos of $TiOSO_4$ solutions. (b) Calibration curve for the reaction of Li_2O_2 and $TiOSO_4$ using the absorbance at 409 nm.

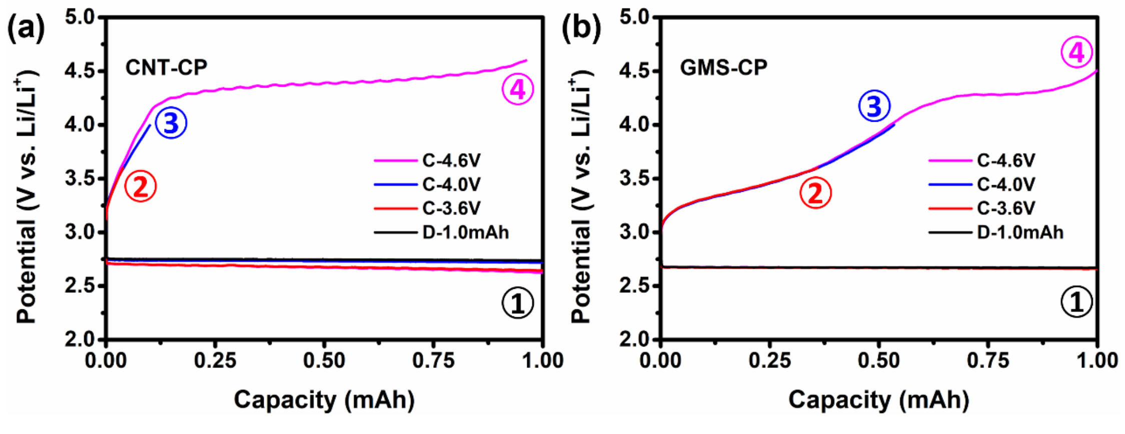

Figure S22 Galvanostatic discharge-charge curves of four Li-O_2 batteries using (a) CNT-CP and (b) GMS-CP in an O_2-saturated 0.5 M LiTFSI/TEGDME under a current of 0.1 mA, a limited capacity of 1.0 mAh, and limited potentials of 3.6 V, 4.0 V, and 4.6 V (vs. Li/Li^+). The four batteries were subjected to discharge-charge tests, and the tests were stopped at different steps. Battery 1: discharged up to 1 mAh (D-1.0mAh).

Batteries 2-4: Discharged up to 1 mAh, and then charged up to 3.6 V (C-3.6V), 4.0 V (C-4.0V), and 4.6 V (C-4.6V), respectively.

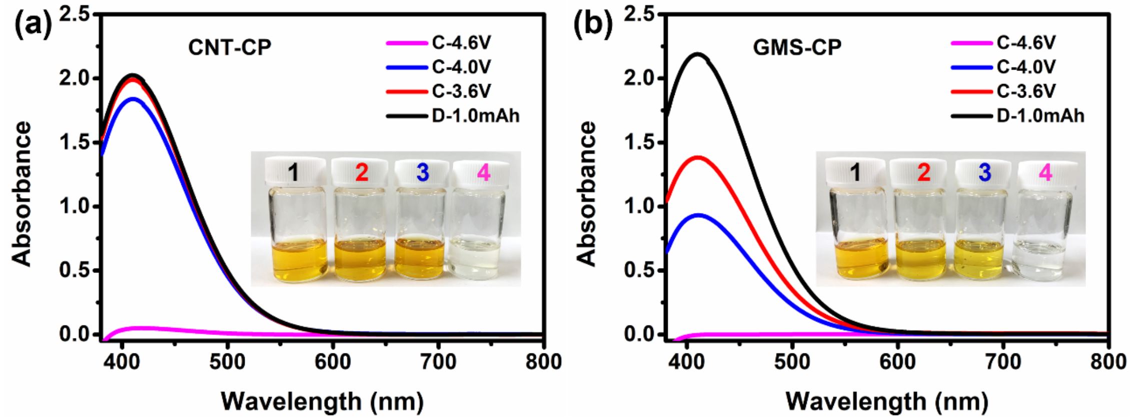

Figure S23 UV-vis spectra of $TiOSO_4$ solutions reacted with (a) CNT-CP and (b) GMS-CP cathodes under different conditions. Insets of a and b show digital photos of $TiOSO_4$ solutions after reacting with cathodes stopped at different conditions. 1: discharged up to 1 mAh (D-1.0mAh). 2-4: Discharged up to 1 mAh, and then charged up to 3.6 V (C-3.6V), 4.0 V (C-4.0V), and 4.6 V (C-4.6V).

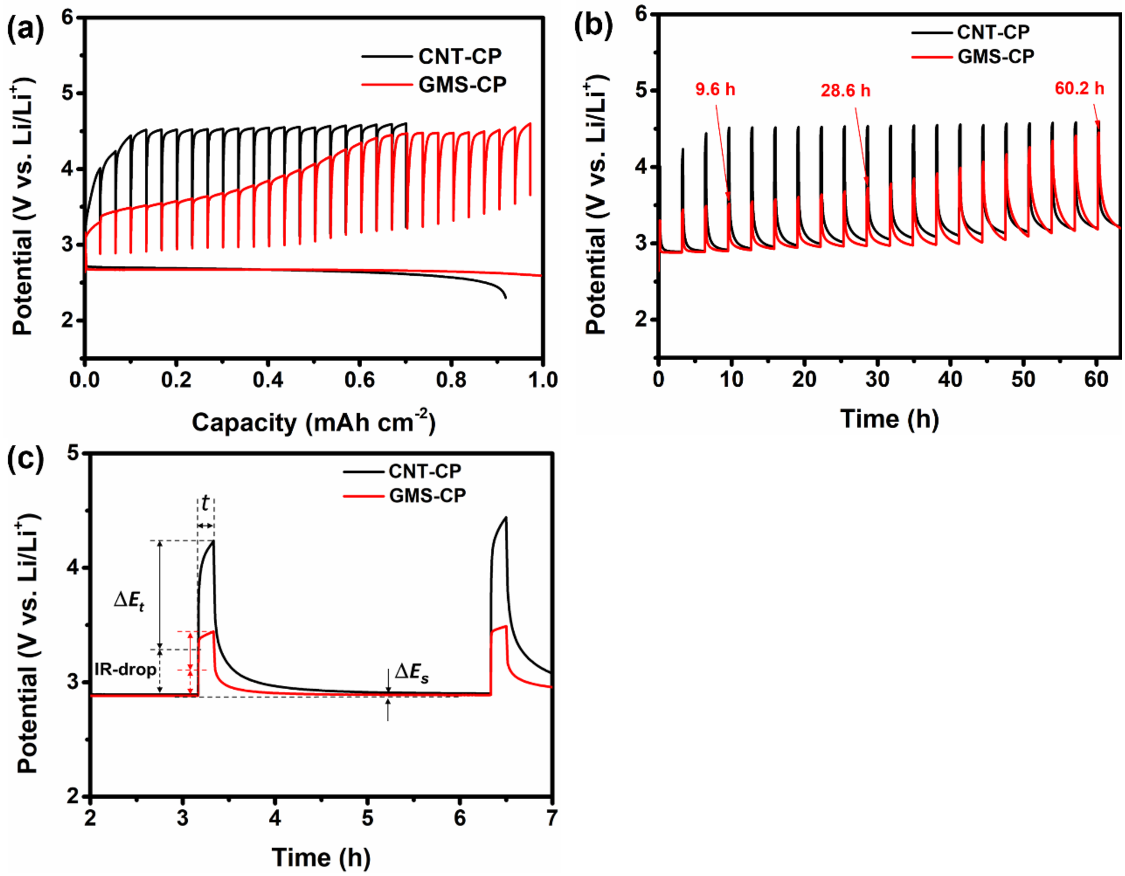

Figure S24 (a) Galvanostatic intermittent titration technique (GITT) of Li-O_2 batteries with a current density of 0.2 mA cm^{-2} for 1.0 mAh cm^{-2}. During GITT measurements, the batteries after discharging were charged by a short pulse of constant current for 10-min and followed by a 3-hours time interval. (b) Time course of E in GITT measurements. (c) The typical cell potential profile of selected areas (2-7 h) from the Figure S24b.

Note S3

Galvanostatic intermittent titration technique (GITT)

The GITT measurement was first developed in 1977 by W. Weppner and R.A. Huggins for the kinetic study of Li-ion in Li_3Sb.[21] Subsequently, this non-destructive technique

has been widely used to study the ion diffusion behavior in Li-ion batteries,[22] and Li-O_2 batteries.[23] As shown in Figure S24a, galvanostatic discharge processes were performed with a limited capacity of 1.0 mAh cm^{-2} and a cut-off potential of 2.3 V (vs. Li/Li^+). Each cycle of the GITT measurement consists of a constant current pulse (0.2 mA cm^{-2} for 10 minutes) and a relaxation period (3 hours). The potential change during the constant current pulses and the relaxation was recorded (Figure S24b). During a constant pulse charging, there is a sudden potential increase in potential from an equilibrium state due to an IR-drop (Figure S24c). The IR-drops were estimated from the changes in cell potential in 60 s immediately after the application of the current pulse. The mobile ions (Li^+) are released from the solid active material, e.g., Li_2O_2, into the liquid electrolyte during the current duration time (t). During the relaxation period, the composition of Li_2O_2 becomes homogeneous by Li^+ diffusion in the Li_2O_2, and the potential reaches another equilibrium state. Based on the potential change (ΔE_t) induced by Li^+ diffusion during the constant current pulse (t), the characteristics of Li^+ diffusion at different charging stages can be investigated, which implies the different charging reaction mechanisms during the charging process.[24] The relationship between the Li^+ diffusion coefficient and the potential (E) and the square root of time ($t^{1/2}$) is represented by the following equation.[21]

$$D_{\mathrm{Li}} = \frac{4}{\pi}\left(\frac{mV_M}{MS}\right)^2\left(\frac{\Delta E_S}{\tau(\frac{dE}{dt^{1/2}})}\right)^2 \qquad \text{(S2)}$$

Where m, V_M, M, and S represent the mass, molar volume, molecular weight of the Li_2O_2, and the surface area of the electrode, respectively.

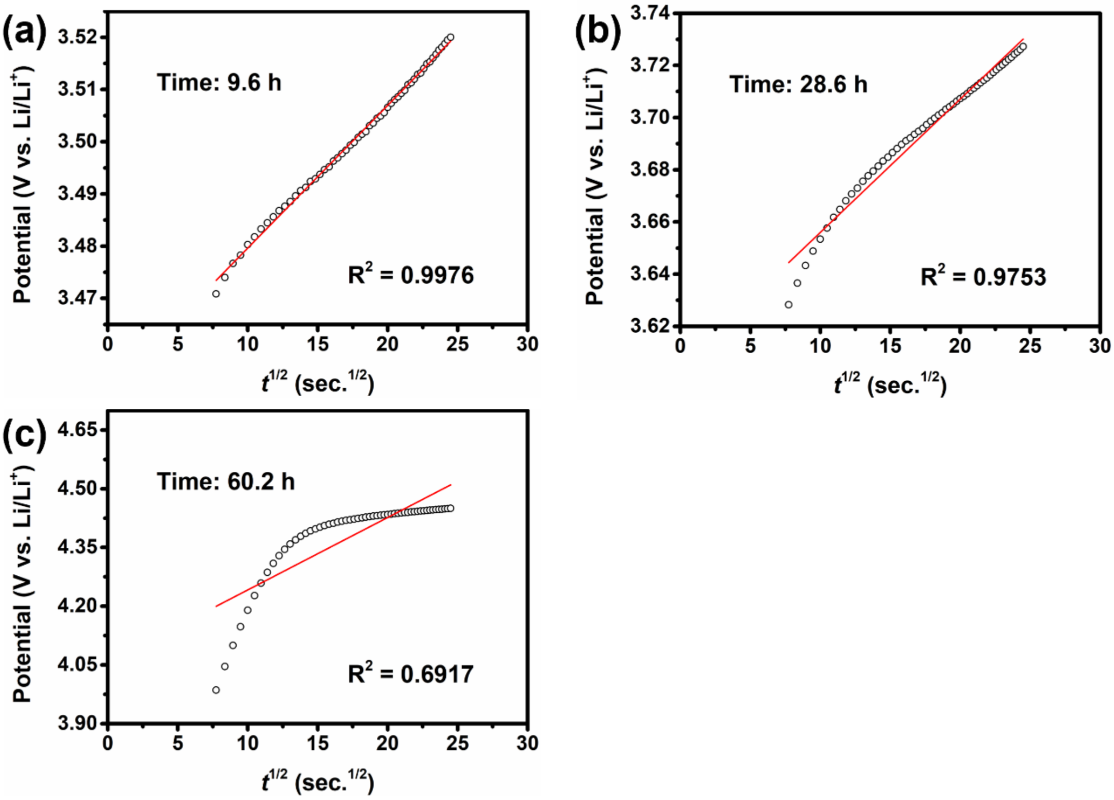

Figure S25 Representative plots of the potential E vs $t^{1/2}$ at (a) 9.6 h, (b) 28.6 h, and (c) 60.2 h in the GITT test of the Li-O_2 battery based on GMS-CP cathode (Figure S24b).

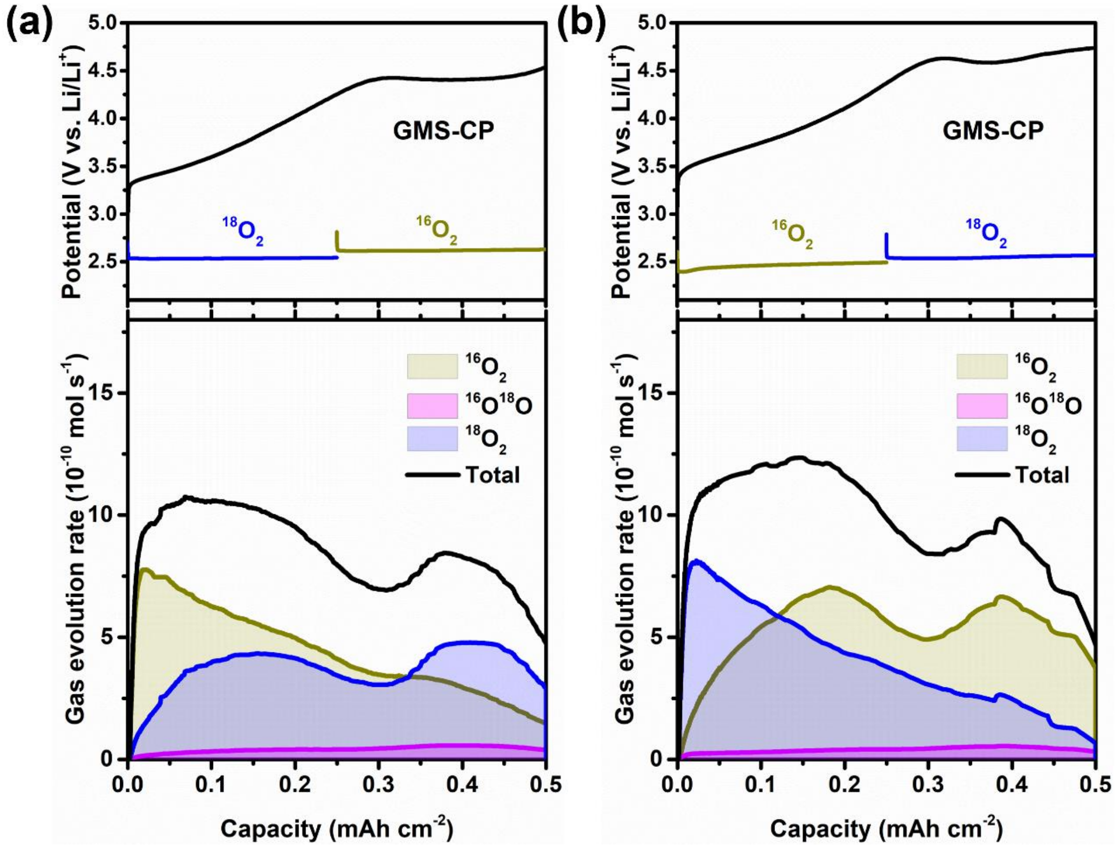

Figure S26 Galvanostatic discharge-charge curves and O_2 evolution rate during the charging process of Li-O_2 batteries in the isotope DEMS test with GMS-CP. (a) Li-O_2 battery was first discharged under $^{18}O_2$ atmosphere with a limited capacity of 0.25 mAh cm^{-2} followed by another discharge process (0.25 mAh cm^{-2}) under $^{16}O_2$ atmosphere ($^{18}O_2$/$^{16}O_2$ supply). (b) Li-O_2 battery was first discharged under $^{16}O_2$ atmosphere with a limited capacity of 0.25 mAh cm^{-2} followed by another discharge process (0.25 mAh cm^{-2}) under $^{18}O_2$ atmosphere ($^{16}O_2$/$^{18}O_2$ supply).

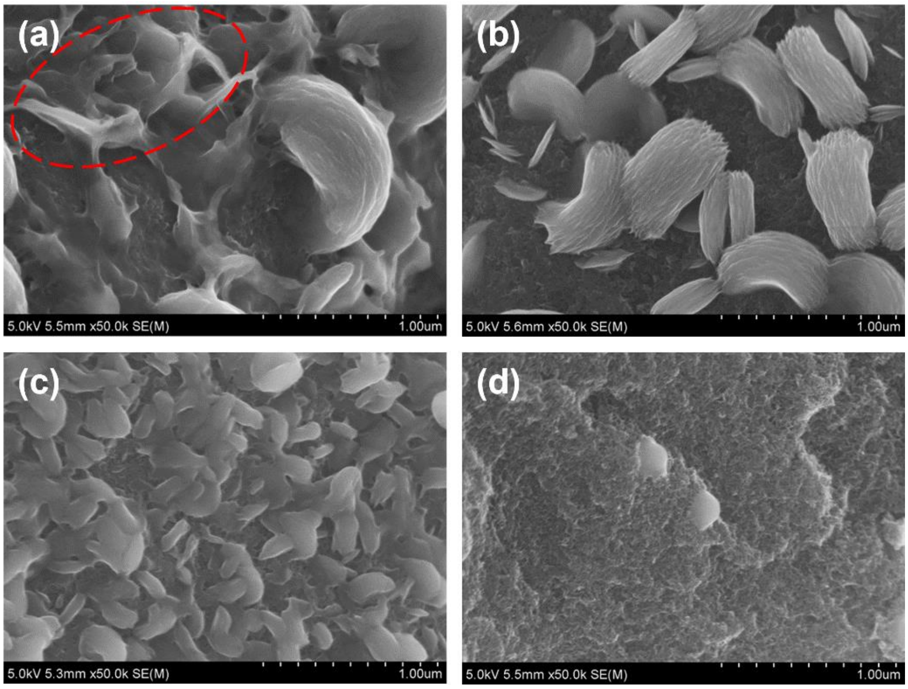

Figure S27 SEM images of GMS-CP cathodes after full-discharge-charge processes. (a) after full-discharging to the limited potential of 2.3 V, (b) after charging to the limited potential of 3.6 V, (c) after charging to the limited potential of 4.0 V, and (d) after full charging to the limited potential of 4.6 V. The red dotted circle in Figure S27a shows the floc-like Li_2O_2 nanosheets. Galvanostatic full-discharge-charge tests of Li-O_2 batteries based on GMS-CP cathode were performed under a current density of 0.2 mA cm^{-2} and a limited potential between 2.3 and 4.6 V (vs. Li/Li^+).

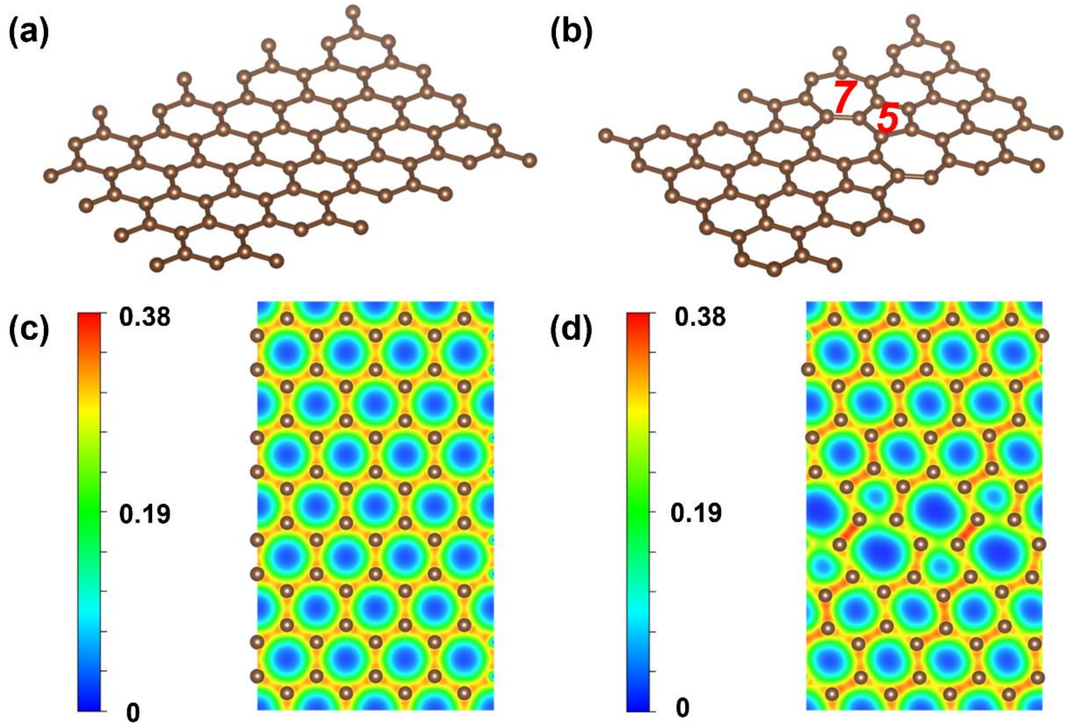

Figure S28 Optimized structures and electron density contours of the (a) graphene with only hexagonal rings and (b) graphene with topological defects (model of GMS). The brown ball represents the carbon atom, and the units of the electron density are |e| (Bohr radius)$^{-3}$.

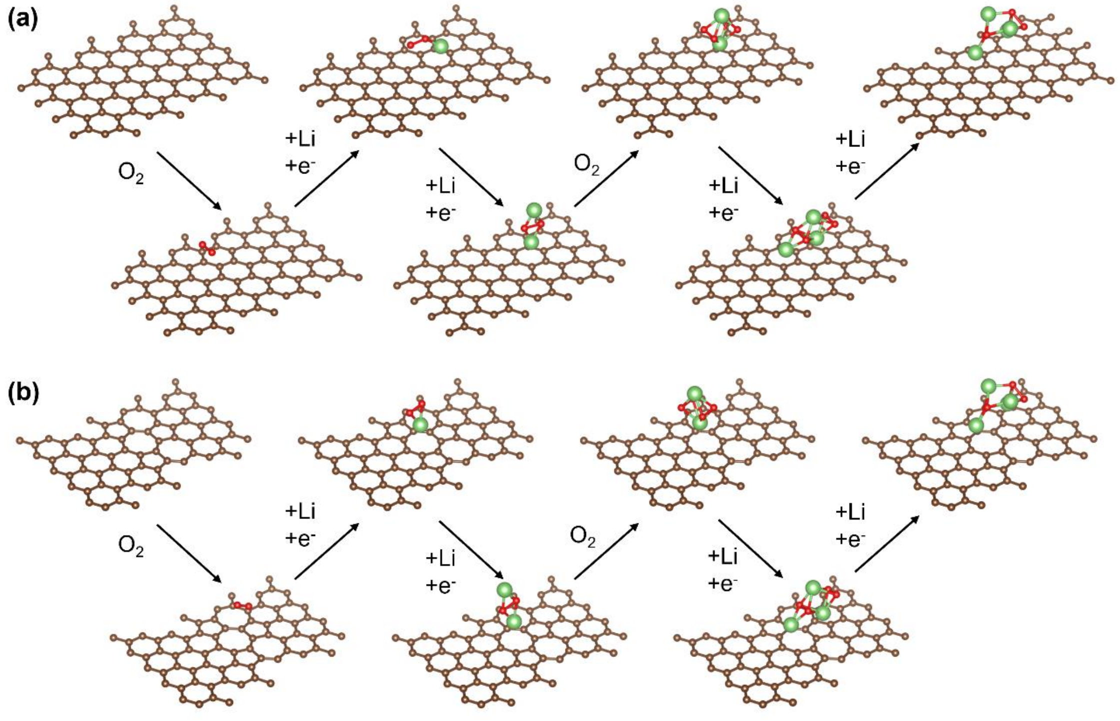

Figure S29 Schematics of Li_4O_4 growing pathways on the (a) graphene with only hexagonal rings and (b) graphene with topological defects. The brown, red, and green balls represent carbon, oxygen, and Li atoms, respectively.

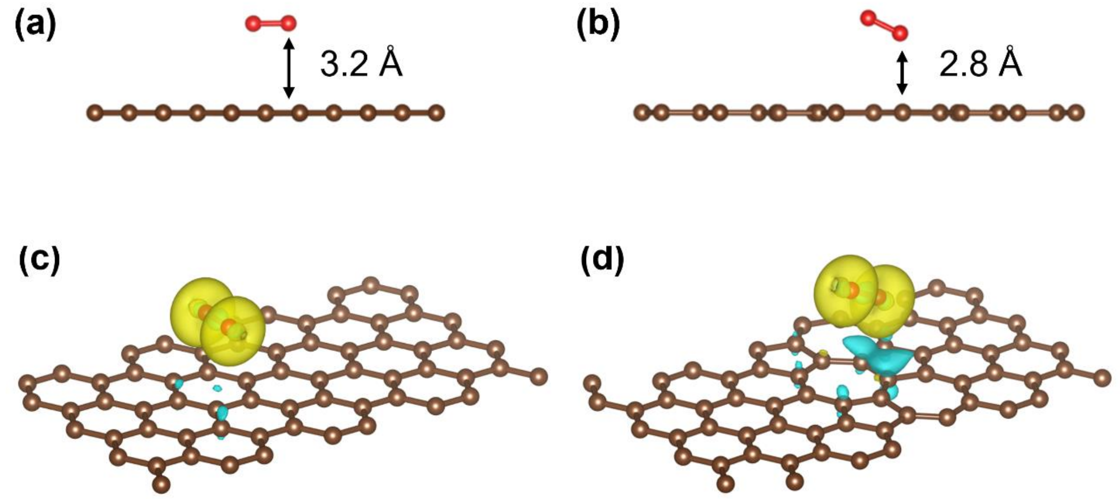

Figure S30 Schematics of O_2 adsorbed on the (a) graphene with only hexagonal rings and (b) graphene with topological defects. Charge difference plots of LiO_2 adsorbed on (c) normal graphene and (d) graphene with topological defects.

Table S1 Cycle performance of Li-O_2 batteries based on a free-standing, pure-carbon cathode without any solid or soluble catalyst.

Carbon material	Electrolyte	Charge potential (V)	Actual Current	Actual Capacity	Cycle number	Cycle life (h)	Ref.
GMS-GO-film	0.5 M LiTFSI in TEGDME	3.6 / 4.1	0.1 mA	0.25 mAh	307	1535	This work
GMS-GO-film	0.5 M LiTFSI in TEGDME	3.6 / 4.2	0.2 mA	0.50 mAh	94	470	This work
GMS-GO-film	0.5 M LiTFSI in TEGDME	3.6 / 4.3	0.4 mA	1.0 mAh	52	260	This work
Well-aligned CNT fibril	1.0 M $LiPF_6$ in TEGDME	4.25	0.015 mA	0.015 mAh	60	120	[25]
Vertically aligned CNTs	0.1 M $LiClO_4$ in TEGDME	4.3	0.1 mA cm^{-2}	2.0 mA cm^{-2}	32	1280	[26]
N-doped graphene aerogels	1 M LiTFSI in TEGDME	4.25	0.144 mA	0.478 mAh	72	480	[27]
Free-standing graphene @g-C_3N_4	1 M $LiCF_3SO_3$ in TEGDME	4.0	0.44 mA	2.2 mAh	105	1050	[28]
Dry-pressed holey graphene	1 M LiTFSI in TEGDME	4.2	0.35 mA	3.5 mAh	20	400	[29]
3D hierarchically porous carbon membrane	1 M LiTFSI in TEGDME	4.0	~ 80 µAh cm^{-2}	32 µA cm^{-2}	40	200	[30]
Nature-inspired GDL/CNF composite	1 M LiTFSI in TEGDME	3.9	500 mA g^{-1}	1000 mAh g^{-1}	32	128	[31]

Table S2 Chemical titration results of the after-discharge and after-charge CNT-CP cathodes of Li-O_2 batteries at different stages.

CNT-CP	Sample 1	Sample 2	Sample 3	Sample 4
**Limited conditions**[a]	D-1.0mAh	C-3.6 V	C-4.0 V	C-4.6V
**Capacity (mAh)**	D-1.0	C-0.039	C-0.100	C-0.962
**Absorbance (counts)**	2.025	1.988	1.838	0.048
**Weight of Li_2O_2 (mg)**[b]	0.617	0.605	0.559	0.013
**Measured ΔW_m (mg)**[c]	/	0.012	0.058	0.604
**Calculated ΔW_c (mg)**[d]	/	0.033	0.086	0.826
**Efficiency (%)**[e]	/	36.4	67.4	73.1

[a] Four CNT-CP cathodes were dissembled from Li-O_2 batteries after discharge or discharge-charge with different limited conditions. Sample 1: CNT-CP after discharge to a limited capacity of 1.0 mAh (D-1.0 mAh), Sample 2: CNT-CP after discharge to a limited capacity of 1.0 mAh and then charge to 3.6 V (C-3.6V), Sample 3: CNT-CP after discharge to a limited capacity of 1.0 mAh and then charge to 4.0 V (C-4.0 V), Sample 4: CNT-CP after discharge to a limited capacity of 1.0 mAh and then charge to 4.6 V (C-4.6 V).

[b] The amount of Li_2O_2 detected by the titration.

[c] The weight decrease of Li_2O_2 during a charging process with a limited potential (3.6 V, 4.0 V, or 4.6 V), determined by the titration. The Li_2O_2 amount (0.617 mg) at discharged to 1.0 mAh is considered as the initial Li_2O_2 amount.

[d] The weight decrease of Li_2O_2 during a charging process with a limited potential (3.6 V, 4.0 V, or 4.6 V), estimated from the charge capacity. The Li_2O_2 amount (0.617 mg) at discharged to 1.0 mAh is considered as the initial Li_2O_2 amount.

[e] The ratio of ΔW_m to ΔW_c.

Table S3 Chemical titration results of the after-discharge and after-charge GMS-CP cathodes of Li-O_2 batteries at different stages.

GMS-CP	Sample 1	Sample 2	Sample 3	Sample 4
**Limited conditions[a]**	D-1.0mAh	C-3.6 V	C-4.0 V	C-4.6V
**Capacity (mAh)**	D-1.0	C-0.359	C-0.534	C-1.0
**Absorbance (counts)**	2.189	1.382	0.930	0
**Weight of Li_2O_2 (mg)[b]**	0.667	0.420	0.282	0
**Measured ΔW_m (mg)[c]**	/	0.247	0.385	0.667
**Calculated ΔW_c (mg)[d]**	/	0.308	0.458	0.859
**Efficiency (%)[e]**	/	80.2	84.1	77.6

[a] Four GMS-CP cathodes were dissembled from Li-O_2 batteries after discharge or discharge-charge with different limited conditions. Sample 1: GMS-CP after discharge

to a limited capacity of 1.0 mAh (D-1.0 mAh), Sample 2: GMS-CP after discharge to a limited capacity of 1.0 mAh and then charge to 3.6 V (C-3.6V), Sample 3: GMS-CP after discharge to a limited capacity of 1.0 mAh and then charge to 4.0 V (C-4.0 V), Sample 4: GMS-CP after discharge to a limited capacity of 1.0 mAh and then charge to 4.6 V (C-4.6 V).

[b] The amount of Li_2O_2 detected by the titration.

[c] The weight decrease of Li_2O_2 during a charging process with a limited potential (3.6 V, 4.0 V, or 4.6 V), determined by the titration. The Li_2O_2 amount (0.667 mg) at discharged to 1.0 mAh is considered as the initial Li_2O_2 amount.

[d] The weight decrease of Li_2O_2 during a charging process with a limited potential (3.6 V, 4.0 V, or 4.6 V), estimated from the charge capacity. The Li_2O_2 amount (0.667 mg) at discharged to 1.0 mAh is considered as the initial Li_2O_2 amount.

[e] The ratio of ΔW_m to ΔW_c.

Table S4 Adsorption energies of Li, O_2, LiO_2, and Li_4O_4 on graphene with only hexagonal rings (6-graphene), GMS with topological defects (5,7-graphene).

	**E_{ads}(Li) (eV)**	**E_{ads}(O_2) (eV)**	**E_{ads}(LiO_2) (eV)**	**E_{ads}(Li_4O_4) (eV)**
**6-Graphene**	1.24	-0.23	0.29	-0.30
**5,7-Graphene**	0.50	-0.28	-0.36	-0.52

Supplementary References

[1] a)H. Nishihara, T. Simura, S. Kobayashi, K. Nomura, R. Berenguer, M. Ito, M. Uchimura, H. Iden, K. Arihara, A. Ohma, Y. Hayasaka, T. Kyotani, *Adv. Funct. Mater.* **2016**, 26, 6418; b)K. Nomura, H. Nishihara, N. Kobayashi, T. Asada, T. Kyotani, *Energy Environ. Sci.* **2019**, 12, 1542; c)H. Nishihara, H.-W. Zhao, K. Kanamaru, K. Nomura, M. Ohwada, M. Ito, L.-X. Li, B.-G. An, T. Horikawa, T. Kyotani, *Carbon Reports* **2022**, 1, 124, 010301; d)H. Nishihara, T. Kyotani, *Adv. Mater.* **2012**, 24, 4473; e)H. Nishihara, T. Kyotani, *Chem. Commun.* **2018**, 54, 5648.

[2] T. Ishii, S. Kashihara, Y. Hoshikawa, J.-i. Ozaki, N. Kannari, K. Takai, T. Enoki, T. Kyotani, *Carbon* **2014**, 80, 135.

[3] K. Nishioka, K. Morimoto, T. Kusumoto, T. Harada, K. Kamiya, Y. Mukouyama, S. Nakanishi, *J. Am. Chem. Soc.* **2021**, 143, 7394.

[4] Y. Shi, *J. Chem. Phys.* **2008**, 128, 234707.

[5] A. P. Thompson, H. M. Aktulga, R. Berger, D. S. Bolintineanu, W. M. Brown, P. S. Crozier, P. J. in 't Veld, A. Kohlmeyer, S. G. Moore, T. D. Nguyen, R. Shan, M. J. Stevens, J. Tranchida, C. Trott, S. J. Plimpton, *Comp. Phys. Comm.* **2022**, 271, 108171.

[6] S. J. Stuart, A. B. Tutein, J. A. Harrison, *J. Chem. Phys.* **2000**, 112, 6472.

[7] a)G. Kresse, J. Hafner, *Phys. Rev. B* **1993**, 48, 13115; b)G. Kresse, J. Furthmüller, *Comp. Mater. Sci.* **1996**, 6, 15.

[8] J. P. Perdew, K. Burke, M. Ernzerhof, *Phys. Rev. Lett.* **1996**, 77, 3865.

[9] a)P. E. Blöchl, *Phys. Rev. B* **1994**, 50, 17953; b)G. Kresse, D. Joubert, *Phys.*

Rev. B **1999**, 59, 1758.

[10] S. Grimme, J. Antony, S. Ehrlich, H. Krieg, *J. Chem. Phys.* **2010**, 132, 154104.

[11] J. Hafner, *J. Mol. Struct.* **2003**, 651-653, 3.

[12] a)P. Giannozzi, S. Baroni, N. Bonini, M. Calandra, R. Car, C. Cavazzoni, D. Ceresoli, G. L. Chiarotti, M. Cococcioni, I. Dabo, A. Dal Corso, S. de Gironcoli, S. Fabris, G. Fratesi, R. Gebauer, U. Gerstmann, C. Gougoussis, A. Kokalj, M. Lazzeri, L. Martin-Samos, N. Marzari, F. Mauri, R. Mazzarello, S. Paolini, A. Pasquarello, L. Paulatto, C. Sbraccia, S. Scandolo, G. Sclauzero, A. P. Seitsonen, A. Smogunov, P. Umari, R. M. Wentzcovitch, *J. Phys.: Condens. Matter* **2009**, 21, 395502; b)P. Giannozzi, O. Andreussi, T. Brumme, O. Bunau, M. Buongiorno Nardelli, M. Calandra, R. Car, C. Cavazzoni, D. Ceresoli, M. Cococcioni, N. Colonna, I. Carnimeo, A. Dal Corso, S. de Gironcoli, P. Delugas, R. A. DiStasio, A. Ferretti, A. Floris, G. Fratesi, G. Fugallo, R. Gebauer, U. Gerstmann, F. Giustino, T. Gorni, J. Jia, M. Kawamura, H. Y. Ko, A. Kokalj, E. Küçükbenli, M. Lazzeri, M. Marsili, N. Marzari, F. Mauri, N. L. Nguyen, H. V. Nguyen, A. Otero-de-la-Roza, L. Paulatto, S. Poncé, D. Rocca, R. Sabatini, B. Santra, M. Schlipf, A. P. Seitsonen, A. Smogunov, I. Timrov, T. Thonhauser, P. Umari, N. Vast, X. Wu, S. Baroni, *J. Phys.: Condens. Matter* **2017**, 29, 465901.

[13] D. Vanderbilt, *Phys. Rev. B* **1990**, 41, 7892.

[14] J. K. Nørskov, J. Rossmeisl, A. Logadottir, L. Lindqvist, J. R. Kitchin, T. Bligaard, H. Jónsson, *J. Phys. Chem. B* **2004**, 108, 17886.

[15] K. Momma, F. Izumi, *J. Appl. Cryst.* **2011**, 44, 1272.

[16] a)R. Tang, M. Yamamoto, K. Nomura, E. Morallón, D. Cazorla-Amorós, H. Nishihara, T. Kyotani, *J. Power Sources* **2020**, 457, 228042; b)S. Sunahiro, K. Nomura, S. Goto, K. Kanamaru, R. Tang, M. Yamamoto, T. Yoshii, J. N. Kondo, Q. Zhao, A. Ghulam Nabi, R. Crespo-Otero, D. Di Tommaso, T. Kyotani, H. Nishihara, *J. Mater. Chem. A* **2021**, 9, 14296.

[17] M. S. Dresselhaus, A. Jorio, R. Saito, *Annu. Rev. Condens. Matter Phys.* **2010**, 1, 89.

[18] a)F. Tuinstra, J. L. Koenig, *J. Chem. Phys.* **1970**, 53, 1126; b)A. C. Ferrari, J. Robertson, *Phys. Rev. B* **2000**, 61, 14095.

[19] a)M. M. Lucchese, F. Stavale, E. H. M. Ferreira, C. Vilani, M. V. O. Moutinho, R. B. Capaz, C. A. Achete, A. Jorio, *Carbon* **2010**, 48, 1592; b)L. G. Cançado, A. Jorio, E. H. M. Ferreira, F. Stavale, C. A. Achete, R. B. Capaz, M. V. O. Moutinho, A. Lombardo, T. S. Kulmala, A. C. Ferrari, *Nano Lett.* **2011**, 11, 3190.

[20] J.-H. Zhong, J. Zhang, X. Jin, J.-Y. Liu, Q. Li, M.-H. Li, W. Cai, D.-Y. Wu, D. Zhan, B. Ren, *J. Am. Chem. Soc.* **2014**, 136, 16609.

[21] W. Weppner, R. A. Huggins, *J. Electrochem. Soc.* **1977**, 124, 1569.

[22] a)Q. Wang, S. Mariyappan, G. Rousse, A. V. Morozov, B. Porcheron, R. Dedryvère, J. Wu, W. Yang, L. Zhang, M. Chakir, M. Avdeev, M. Deschamps, Y.-S. Yu, J. Cabana, M.-L. Doublet, A. M. Abakumov, J.-M. Tarascon, *Nat. Mater.* **2021**, 20, 353; b)Y. Zhu, C. Wang, *J. Phys. Chem. C* **2010**, 114, 2830.

[23] a)J. Zhang, Y. Zhao, B. Sun, Y. Xie, A. Tkacheva, F. Qiu, P. He, H. Zhou, K. Yan, X. Guo, S. Wang, M. McDonagh Andrew, Z. Peng, J. Lu, G. Wang, *Sci. Adv.* **2022**, 8, 1899; b)Y. Hase, T. Uyama, K. Nishioka, J. Seki, K. Morimoto, N. Ogihara, Y. Mukouyama, S. Nakanishi, *J. Am. Chem. Soc.* **2022**, 144, 1296; c)Z. H. Cui, X. X. Guo, H. Li, *Energy Environ. Sci.* **2015**, 8, 182.

[24] a)Y.-C. Lu, Y. Shao-Horn, *J. Phys. Chem. Lett.* **2013**, 4, 93; b)B. M. Gallant, D. G. Kwabi, R. R. Mitchell, J. Zhou, C. V. Thompson, Y. Shao-Horn, *Energy Environ. Sci.* **2013**, 6, 2518.

[25] H.-D. Lim, K.-Y. Park, H. Song, E. Y. Jang, H. Gwon, J. Kim, Y. H. Kim, M. D. Lima, R. O. Robles, X. Lepró, R. H. Baughman, K. Kang, *Adv. Mater.* **2013**, 25, 1348.

[26] S. Huang, W. Fan, X. Guo, F. Meng, X. Liu, *ACS Applied Materials & Interfaces* **2014**, 6, 21567.

[27] C. Zhao, C. Yu, S. Liu, J. Yang, X. Fan, H. Huang, J. Qiu, *Adv. Funct. Mater.* **2015**, 25, 6913.

[28] W.-B. Luo, S.-L. Chou, J.-Z. Wang, Y.-C. Zhai, H.-K. Liu, *Small* **2015**, 11, 2817.

[29] Y. Lin, B. Moitoso, C. Martinez-Martinez, E. D. Walsh, S. D. Lacey, J.-W. Kim, L. Dai, L. Hu, J. W. Connell, *Nano Lett.* **2017**, 17, 3252.

[30] S.-M. Xu, X. Liang, Z.-C. Ren, K.-X. Wang, J.-S. Chen, *Angew. Chem. Int. Ed.* **2018**, 130, 6941.

[31] M.-C. Kim, J.-Y. So, S.-H. Moon, S.-B. Han, S. Choi, E.-S. Kim, Y.-K. Shin, J.-E. Lee, D.-H. Kwak, C. Lee, W.-G. Bae, K.-W. Park, *J. Mater. Chem. A* **2018**, 6, 9550.
